# Germplasm resources and genetic breeding of *Paeonia*: a systematic review

**DOI:** 10.1038/s41438-020-0332-2

**Published:** 2020-07-01

**Authors:** Yong Yang, Miao Sun, Shanshan Li, Qihang Chen, Jaime A. Teixeira da Silva, Ajing Wang, Xiaonan Yu, Liangsheng Wang

**Affiliations:** 1 Key Laboratory of Plant Resources/Beijing Botanical Garden, Institute of Botany, Chinese Academy of Sciences, 100093 Beijing, China; 2 College of Landscape Architecture, Beijing Forestry University, 100083 Beijing, China; 3 Beijing Key Laboratory of Ornamental Plants Germplasm Innovation and Molecular Breeding, 100083 Beijing, China; 4 National Engineering Research Center for Floriculture, 100083 Beijing, China; 5 University of Chinese Academy of Sciences, 100049 Beijing, China; 6 P.O. Box 7, Miki–cho post office, Ikenobe 3011–2, Kagawa–ken, 761–0799 Japan

**Keywords:** Plant breeding, Genetic hybridization

## Abstract

Members of the genus *Paeonia*, which consists of globally renowned ornamentals and traditional medicinal plants with a rich history spanning over 1500 years, are widely distributed throughout the Northern Hemisphere. Since 1900, over 2200 new horticultural *Paeonia* cultivars have been created by the discovery and breeding of wild species. However, information pertaining to *Paeonia* breeding is considerably fragmented, with fundamental gaps in knowledge, creating a bottleneck in effective breeding strategies. This review systematically introduces *Paeonia* germplasm resources, including wild species and cultivars, summarizes the breeding strategy and results of each *Paeonia* cultivar group, and focuses on recent progress in the isolation and functional characterization of structural and regulatory genes related to important horticultural traits. Perspectives pertaining to the resource protection and utilization, breeding and industrialization of *Paeonia* in the future are also briefly discussed.

## Introduction

The genus *Paeonia* was placed in the Ranunculaceae before the 20th century. According to the characteristic arrangement of stamens, Worsdell^1^ separated *Paeonia* from rose in the Ranunculaceae and moved it to the Paeoniaceae. *Paeonia* species or cultivars are shrubs or perennial herbs. Wild *Paeonia* species are mainly distributed in temperate regions of the Northern Hemisphere, although individual species extend into cold temperate regions, with latitudes ranging from 24.4 to 66.5°N^2,3^. Wild woody *Paeonia* species are distributed only in China, whereas herbaceous species are widely distributed throughout Central and East Asia, the Himalayas, western North America (USA and Canada), and the Mediterranean^4^. These widely different habitats have led to very rich genetic variation in *Paeonia*.

Paeoniflorin and paeonol are unique chemical components in the roots of *Paeonia*, so several species of *Paeonia* were initially utilized as medicinal plants^5^. Wild *Paeonia* species were introduced and domesticated in China approximately 1500 years ago, as well as in medieval Europe^6,7^. Members of the genus *Paeonia* were first used as ornamental plants in China, and then this cultural use spilled over into other East Asian countries such as Japan and South Korea^8^. In the 18th century, Chinese tree and herbaceous peony cultivars were introduced into Europe, and later, into North America^9,10^. These *Paeonia* cultivars were very popular, spread widely throughout Europe and North America, and led to considerable cross-breeding work in the 19th century. The subsequent explosion of new *Paeonia* cultivars laid a foundation for the industrialization of *Paeonia*. In recent years, herbaceous peony has been used as a new cut flower^7,11,12^, and several major cut flower production areas have formed around the world, including in China, the Netherlands, North America, and New Zealand^13^. In 2011, the Ministry of Health of the People’s Republic of China approved tree peony seed oil as a new food resource, so the scale of oil tree peony planting has expanded rapidly in recent years. A review of oil tree peony and *Paeonia* medicinal plants will be published separately.

With the rapid development of *Paeonia* industrialization around the world, an increasing number of excellent cultivars are urgently needed. However, there are currently many problems in *Paeonia* breeding, such as an incomplete understanding of the extent of germplasm resources, rudimentary breeding techniques, fragmentary breeding information, unclear breeding objectives, and low breeding efficiency, among other limitations. This review provides a summary of the germplasm resources and genetic breeding of *Paeonia*, focusing primarily on the classification and characteristics of wild species and cultivar resources, phenotypic inheritance, molecular marker-assisted breeding, breeding methods and their applications, molecular breeding, and some prospects for breeding in the future. An important purpose of this review is to provide ideas and inspiration for *Paeonia* breeders. The method used for literature retrieval and selection for coverage is described in Supplementary Data 1.

## Germplasm resources

### Wild species resources

Linnaeus^14^ described the genus *Paeonia* for the first time based on two specimens from Switzerland and named one species *Paeonia officinalis* L., with two varieties, *feminea* and *mascula*. According to a cultivar of *Paeonia* introduced to Europe from Guangzhou, China, in 1794, Andrews^15^ introduced the first tree peony plant and named it *Paeonia suffruticosa*. In 1818, Anderson published the first monograph of *Paeonia*, which included 13 species divided into two major species groups, Fruticosa and Herbaceae^16^. The first *Paeonia* species of the New World (North America) was recorded by Douglas but later published by Hooker^17^ and named *Paeonia brownii*. Since then, *Paeonia* species all over the world have been systematically recognized. In 1890, Lynch reclassified *Paeonia* into three subgenera, *Moutan*, *Onaepia*, and *Paeonia*^18^. Following Lynch’s opinion^18^ but using the lower rank of section instead of subgenus, Stern^19^ divided the genus into three major groups: sect. *Moutan*, including all woody peonies; sect. *Onaepia*, including all herbaceous peonies in the New World; and sect. *Paeon* (=*Paeonia*), including all herbaceous peonies in the Old World (Table 1). Stern’s view was widely accepted by later scholars^7,20^, and taxonomic studies of *Paeonia* that employed molecular biology also support Stern’s view^4,21–24^.

**Table 1:** Species of *Paeonia* their chromosome number

Section	Subsection	Species and their chromosome number
I. *Onaepia*		*P. brownii* Douglas ex Hook. (2n = 10), *P. californica* Nutt. ex Torr. & A. Gray (2n = 10)
II. *Moutan*	*Vaginatae*	*P. decomposita* Hand.-Mazz. (2n = 10), *P. rotundiloba* (D. Y. Hong) D. Y. Hong (2n = 10), *P. jishanensis* T. Hong & W. Z. Zhao (2n = 10), *P. ostii* T. Hong & J. X. Zhang (2n = 10), *P. qiui* Y. L. Pei & D. Y. Hong (2n = 10), *P. rockii* subsp. *atava* (Brühl) D. Y. Hong & K. Y. Pan (2n = 10), *P. rockii* subsp. *rockii* (S. G. Haw & L. A. Lauener) T. Hong & J. J. Li ex D. Y. Hong (2n = 10), *P*. × *baokangensis* Z. L. Dai & T. Hong (2n = 10), *P*. × *yananensis* T. Hong & M. R. Li (2n = 10), *P. × suffruticosa* Andrews (2n = 10)
	*Delavayanae*	*P. delavayi* Franch. (2n = 10), *P. lutea* Delavay ex Franch. (2n = 10), *P. potaninii* Kom. (2n = 10), *P. ludlowii* (Stern & G. Taylor) D. Y. Hong (2n = 10)
III. *Paeonia*	*Albiflorae*	*P. anomala* L. (2n = 10), *P. veitchii* Lynch (2n = 10), *P. emodi* Wall. ex Royle (2n = 10 or 20), *P. lactiflora* Pall. (2n = 10), *P. sterniana* H. R. Fletcher (2n = 10)
	*Foliatae*	*P. algeriensis* (2n = ?), *P. broteri* Boiss. & Reut. (2n = 10 or 20), *P. cambessdesii* (Willk.) Willk. (2n = 10), *P. clusii* subsp. *clusii* Stern (2n = 10 or 20), *P. clusii* subsp. *rhodia* (Stearn) Tzanoud. (2n = 10), *P. coriacea* Boiss. (2n = 20), *P. corsica* Sieber ex Tausch (2n = 10), *P. daurica* subsp. *velebitensis* D. Y. Hong (2n = ?), *P. daurica* subsp. *macrophylla* (Albov) D. Y. Hong (2n = 20), *P. daurica* subsp. *wittmanniana* (Hartwiss ex Lindl.) D. Y. Hong (2n = 20), *P. daurica* subsp. *mlokosewitschii* (Lomakin) D. Y. Hong (2n = 10), *P. daurica* subsp. *daurica* Andrews (2n = 10), *P. daurica* subsp. *coriifolia* (Rupr.) D. Y. Hong (2n = 10), *P. daurica* subsp. *tomentosa* (Lomakin) D. Y. Hong (2n = 20), *P. kesrouanensis* (Thiébaut) Thiébaut (2n = 20), *P. mairei* Pall. (2n = 10 or 20), *P. mascula* subsp. *mascula* Stearn & Davis (2n = 20), *P. mascula* subsp. *russio* (Biv.) Cullen & Heywood (2n = 20), *P. mascula* subsp. *bodurii* N. Özhatay (2n = 20), *P. mascula* subsp. *hellenica* Tzanoud. (2n = 20), *P. obovata* subsp. *obovata* Maxim. (2n = 10 or 20), *P. obovata* subsp. *willmottiae* (Stapf) D. Y. Hong & K. Y. Pan (2n = 20)
	*Paeonia*	*P. arietina* G. Anderson (2n = 20), *P. intermedia* C. A. Mey. (2n = 10), *P. parnassica* (Boiss. & Reut.) Nym. (2n = 20), *P. peregrine* Mill. (2n = 20), *P. saueri* D. Y. Hong, X. Q. Wang & D. M. Zhang (2n = 20), *P. tenuifolia* L. (2n = 10), *P. officinalis* subsp. *microcarpa* (Boiss. & Reut.) Nym. (2n = 20), *P. officinalis* subsp. *banatica* (Rochel) Soó (2n = 20), *P. officinalis* subsp. *huthii* Soldano (2n = 20), *P. officinalis* subsp. *italica* Passalacqua & Bernardo (2n = 20), *P. officinalis* subsp. *officinalis* L. (2n = 20), *P*. × *saundersii* Stebbins (2n = 10)

According to refs. ^2,4–6,24,39,40^ and field surveys

#### Section Moutan

**Fig. 1:**
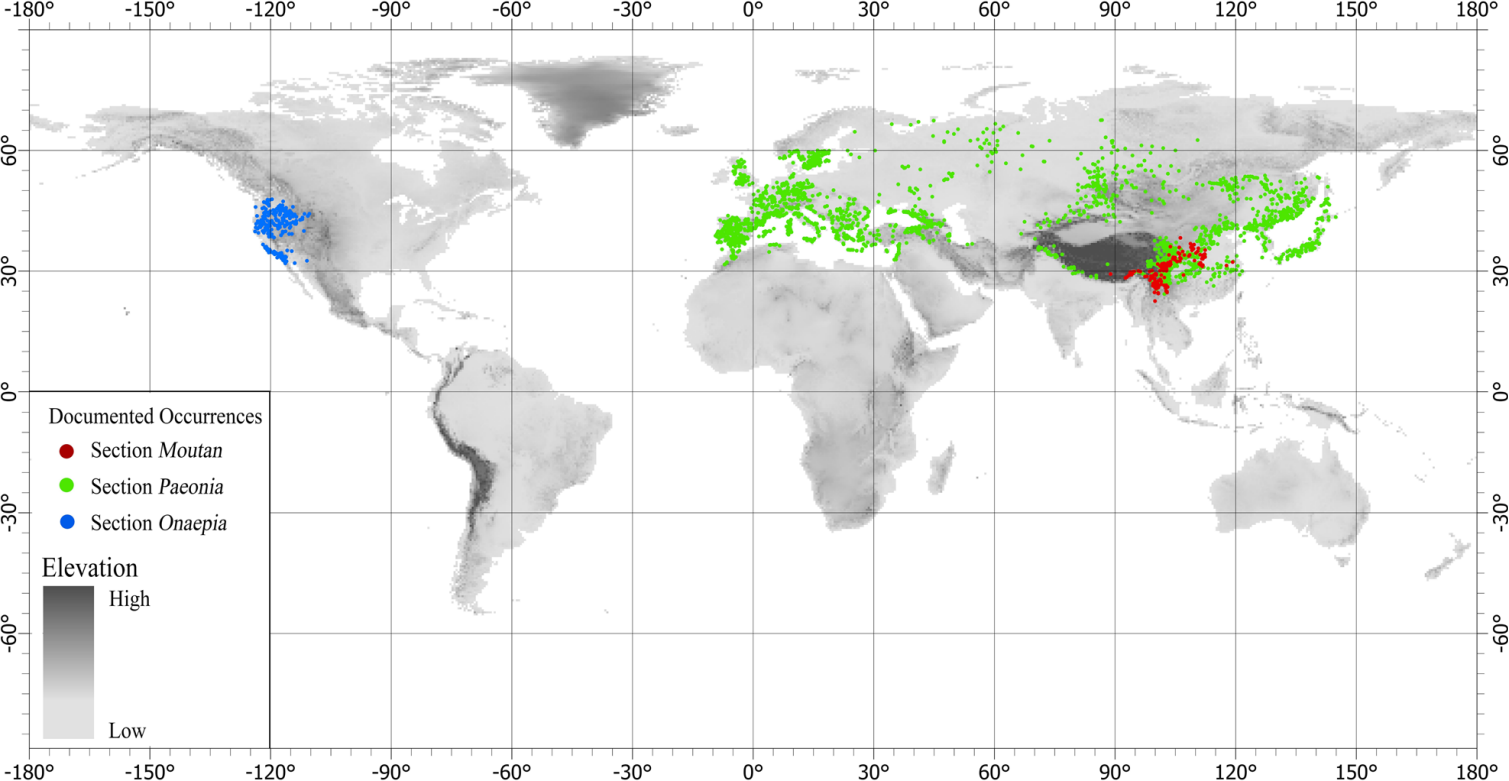
Geographical distribution of wild species of *Paeonia*. Dots on the map indicate the locations where wild species of *Paeonia* were collected. Red dots indicate section *Moutan*, green dots indicate section *Paeonia*, and blue dots indicate section *Onaepia*

All species of sect. *Moutan*, which are endemic to China (Fig. 1), are shrubs or subshrubs, with 2n = 10 ^4^. According to morphological differences of the disk, sect. *Moutan* was divided into two subsections. The species with a thin leathery sheath that at first completely envelops the carpels belong to subsect. *Vaginatae*, while species with conspicuous fleshy lobes around the base of carpels belong to subsect. *Delavayanae*^24^. Species of sect. *Moutan* have been continuously discovered over the past 100 years^2,20,25–36^. In 1999, Hong and Pan^37,38^ systematically sorted out the species of sect. *Moutan*. They reported eight species in this section, three of which included two separate varieties. In the *Flora of China*, Hong et al.^39^ revised this group to eight species and two subspecies. Hong^40^ then elevated the two subspecies of *Paeonia decomposita* into two separate species, but with no change in the other species (Supplementary Data 2).

Hong’s taxonomic work on sect. *Moutan* is systematic and comprehensive. Although the vast majority of the taxonomy in sect. *Moutan* is widely accepted by other scholars, a few different views exist about select species. Hong and Pan^41^ named a new species, *Paeonia cathayana*, based on a tree peony found at a farmer’s home in Henan Province, China. The owner claimed that the tree peony was dug from the wild, although no natural populations of this species were found in the wild in a subsequent investigation. Hong et al.^36^ believed that morphological variation is continuous among *Paeonia delavayi*, *Paeonia lutea* and *Paeonia potaninii*, treating them as single species and without any subspecies. Zhang^42^ studied the genetic diversity of the *P. delavayi* complex (including *P. delavayi*, *P. lutea*, and *P. potaninii*) from 14 populations and found large genetic differentiation among populations in different regions. Based on a years-long field investigation and experience in introduction and domestication, Li et al.^6^ believed that these three species had great differences in morphology and that these differences were stable among offspring. According to their results, Li et al. felt that the types within *P. delavayi* should be distinguished to allow for their more effective protection and utilization. For simplicity, we treat them as three species in this paper.

Hong and Pan^43^ claimed that “*P. suffruticosa* is not a hybrid”. However, many scholars have confirmed that *P. suffruticosa* is a hybrid formed by repeated hybridization of several species of subsect. *Vaginatae*, based on morphological and molecular biological evidence^5,44–47^. Therefore, we suggest changing the name of this species to *Paeonia* × *suffruticosa*.

#### Section *Paeonia*

The species of sect. *Paeonia* are perennial herbs that are widely distributed in East and Central Asia, the Himalayas, and the Mediterranean region (Fig. 1) and have 2n = 10 or 20^48^. Due to polyploidization and reticulate evolution, many different groups formed in the Mediterranean, making their classification very difficult^48,49^.

According to the number of leaflets or segments of lower leaves, sect. *Paeonia* was once divided into two subsections, subsect. *Foliolatae* and subsect. *Dissectifoliae*^24^. Beginning in the 1990s, Hong et al.^50–59^ carefully investigated sect. *Paeonia* distributed in Eurasia and the Mediterranean, studied its type specimens, and then revised the classification of sect. *Paeonia* comprehensively and systematically. They used the number of flowers per stem and root morphology as the main classification indexes and divided this section into three subsections: subsect. *Albiflorae* (usually several flowers per stem, root more or less carrot-shaped), subsect. *Foliolatae* (flower always solitary and terminal, roots carrot-shaped) and subsect. *Paeonia* (flowers solitary and terminal, lateral roots fusiform or tuberous), including 22 species and 22 subspecies (Supplementary Data 2)^24^.

In the *Flora of China*, *Paeonia veitchii* and *Paeonia anomala* are listed as separate species^39^. Hong and Pan^55^ deemed these species to have similar external morphologies but a difference in the number of flowers per stem, treating them as two subspecies of *P. anomala*. However, molecular evidence (nuclear and chloroplast genetic markers) indicated that the genetic distance between the two species was far, while *P. veitchii* and *Paeonia sternadia* were more closely related^48^. In the classification of *Paeonia*, the characteristics of seeds are hardly considered, even though there are great differences between species. We compared the seeds of *P. veitchii* and *P. anomala* and found that the testa of *P. veitchii* was dark blue and fleshy, while that of *P. anomala* was black and nonfleshy. Based on our assessment and on work conducted by Xia^48^, it is suggested that the two species should be independent (Yang et al., 2020, unpublished data). Hong^4,40^ made a breakthrough in the classification of sect. *Paeonia*. However, the classification of the three subsections was not completely consistent with a phylogenetic tree constructed by other scholars using nuclear and chloroplast genetic markers. *Paeonia* molecular phylogenetic analyses^4,21,23,48,60^ revealed that even the parents of different subspecies within the same species were completely different. All these results indicate that the relationships within sect. *Paeonia* are complex and that its classification requires additional studies.

#### Section *Onaepia*

There are only two species (*P. brownii* and *Paeonia californica*) in sect. *Onaepia*, with 2n = 10 ^4^. They are perennial herbs with lateral roots that are carrot-shaped, fusiform or tuberous. The flowers are solitary and terminal or several per stem. The short petals are slightly longer or shorter than the sepals, and the disk is annular and fleshy, enveloping only the base of the carpels^4^. The distribution areas of the two species mostly overlap: *P. californica* is limited to the south of California, while *P. brownii* is distributed from northern California to Washington (Fig. 1). The flowering time of *P. californica* is from February to April, while that of *P. brownii* is from June to July^61^. These two species have very similar morphologies. They were regarded as a single species until a series of detailed morphological, ecological, and cytogenetic studies showed that they are different^62^. DNA sequencing also supports the above conclusion, lending evidence that the molecular evolution in this section was significantly faster than its morphological evolution^22^.

### Cultivar resources

*Paeonia* was first domesticated and cultivated in China, including *P*. × *suffruticosa* and *Paeonia lactiflora* cultivars^5,63^. In the 18th century, Chinese tree peony and herbaceous peony cultivars were introduced into Europe and then spread to many temperate regions around the world^9^. With the spread of Chinese peony cultivars, different cultivated groups of *Paeonia* have gradually formed worldwide.

#### Tree peony cultivars

**Fig. 2:**
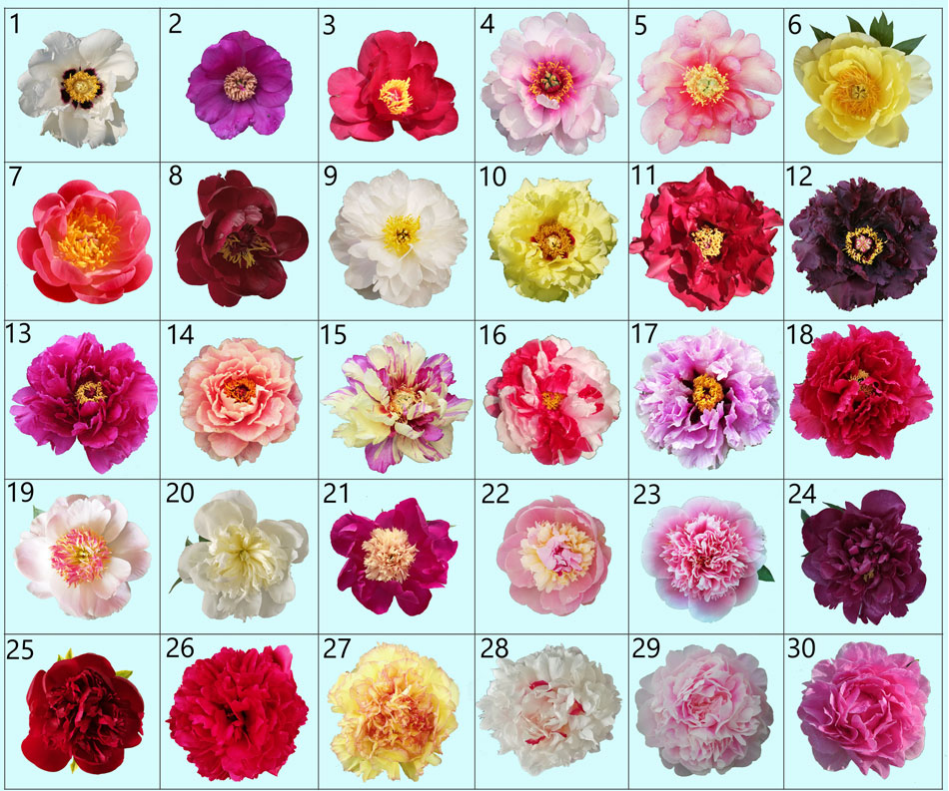
Typical flower colors and types in *Paeonia*. 1–3, single type; 4–18, semi-double type; 19–22, Japanese type; 23–26, bomb type; and 27–30, full double type. The taxonomy and name of cultivars (species) are shown in Supplementary Data 3

Wild tree peony may have been introduced and domesticated in China during the Wei and Jin Dynasties. After nearly a thousand years of development, it formed four large cultivar groups (Zhongyuan, northwest, southwest, and Jiangnan) and several smaller cultivar groups, such as the Yan’an and Baokang groups^5,6,63^. Zhou et al.^47^ used 14 chloroplast gene fragments and 25 single-copy nuclear genes to study the origin of *P*. × *suffruticosa*, concluding that *Paeonia rockii*, *Paeonia qiui*, *Paeonia ostii*, *Paeonia jishanensis* and *P. cathayana* participated in the formation of traditional *P*. × *suffruticosa*, while *P. decomposita* and subsect. *Delavayanae* did not. This conclusion was mostly consistent with those of other studies^46,63–65^. At present, there are more than 1000 tree peony cultivars in China, producing white, pink, red, purple, black, violet, and multicolored flowers with single, half, double and other flower types (Fig. 2)^6,11^.

Chinese tree peony cultivars were introduced to Japan starting in the 8th century and largely in the 16th century. In the following two centuries, tree peony was bred independently to meet Japanese esthetic preferences and local climate characteristics on the basis of Chinese tree peony. Japanese tree peony cultivars have distinct characteristics. Most of them produce single or semidouble flowers with bright red or purple colors, beautiful lines, and an upright flower that is higher than the leaf surface. Approximately 200 tree peony cultivars have been bred in Japan^8,66^.

Most of the early tree peony cultivars in Europe were domesticated from Chinese tree peony^8,9^. At the end of the 19th century, *P. delavayi* and *P. lutea* were found in Yunnan, China^25^, and then introduced to Europe and North America. A series of cultivars were bred by crossing the two wild species with *P*. × *suffruticosa* in France and the USA^9^. The flower colors of this group of tree peonies include yellow, champagne, orange, and scarlet, which do not exist in *P*. × *suffruticosa*. The flower stems are not strong, and some cultivars have drooping flowers. They flower towards the end of spring, a week or two after Chinese or Japanese tree peonies. These hybrid cultivars, which were formed by crossing *P. delavayi* and *P. lutea* with *P*. × *suffruticosa* or their offspring, interbreed and are classified into the Lutea hybrid group. There are more than 1000 cultivars, mainly in the USA, Europe and Australasia^9,11,67,68^.

#### Herbaceous peony cultivars

The earliest record of cultivation of ornamental herbaceous peonies in China was also during the Wei and Jin Dynasties^69,70^. *P. lactiflora* is widely distributed in China and has strong adaptability. Chinese herbaceous peony cultivars were bred by domesticating this single species^71^. Therefore, the cultivars of this group are called Lactiflora or Chinese peony^9^. There are ~300 cultivars of Lactiflora peony in China, including cultivars producing white, pink, red, purple, purple-black, multicolored, and other-colored flowers (Fig. 2). The flower types include the single, semidouble, double, Japanese and bomb types^5,12^.

Lactiflora peony was introduced into Japan in the 10th century. Japanese breeders began breeding this peony in the 17th century, creating ~100 cultivars to date^66^. In the 19th century, Lactiflora peony cultivars became popular in Europe and North America, when much breeding work was carried out. After more than 100 years of development, Lactiflora peony cultivars have developed into the largest group of *Paeonia* in Europe and North America, with more than 4000 cultivars registered with the American Peony Society (APS)^9,68^. At the end of the 19th century, European and American breeders attempted to use Lactiflora peonies in crosses with European wild peony species^10^. These kinds of hybrid cultivars, which are produced by crossing two or more species of sect. *Paeonia*, are called herbaceous hybrid peonies. The flowering time of herbaceous hybrid peonies is usually earlier than that of Lactiflora peonies, which greatly prolongs their entire flowering period. They also contribute new flower colors, such as yellow, coral, and bright red, which are not available in Lactiflora peonies (Fig. 2)^7^. At present, there are more than 1000 herbaceous hybrid peonies registered with the APS, and the number is increasing^68^.

#### Intersectional hybrid cultivars

The success of intersectional *Paeonia* hybrids is a tremendous breakthrough in the contemporary history of *Paeonia* breeding. Japanese breeders successfully hybridized herbaceous peony with tree peony, which was widely developed by American breeders. Thus far, only sect. *Paeonia* and sect. *Moutan* have been successfully hybridized among the three *Paeonia* sections^72^. At present, most cultivars are obtained by using Lactiflora peonies as the female parent and Lutea hybrid peonies as the male parent. To commemorate the pioneering contribution of the Japanese breeder Toichi Itoh in peony intersectional breeding, the APS refers to these cultivars, which were obtained from the Lactiflora peony (♀) × Lutea Hybrid peony (♂) cross (♀ = female parent, ♂ = male parent), as Itoh hybrid peonies. The flower and leaf types of intersectional hybrid peonies are mostly similar to those of tree peonies, and their ecological habits and flowering times are similar to those of herbaceous peonies. Most intersectional hybrid peonies have strong adaptability, few diseases, and abundant flowers. Their flower colors are rich, including white, yellow, pink, orange, purple, and scarlet (Fig. 2)^73^. More than 150 cultivars have been registered^68^.

### Horticultural utilization of *Paeonia*

The application of peony cultivars mainly focuses on three aspects: landscape architecture, potted plants and cut flowers. Special peony gardens are most famous in China and Japan. In Luoyang, Heze and Beijing, China, and Shimane, Japan, there are more than 30 large special peony gardens, with the largest number in Luoyang^6^. Each year, more than 20 million tourists visit Luoyang during the flowering period, generating total tourism-related revenue that exceeds 17.8 billion yuan (approximately 2.52 billion US$)^74^. In Europe, North America and other countries, peony cultivars are mainly used for landscape gardening.

Peony cultivars are widely sold as potted plants in Japan, Europe and North America, greatly prolonging the sales period of peony plants. In China, potted tree peonies blooming during the Spring Festival are the most common. In Heze, the annual production of potted tree peonies exceeds 290 thousand pots during the Spring Festival, and the output value exceeds 30 million yuan (Heze Daily, 25 December 2019).

On the international flower market, herbaceous peony is becoming popular as a new cut flower. More than 25 countries are engaged in the production of herbaceous peony cut flowers, with the Netherlands being the largest global producer, annually producing more than 48 million herbaceous peony cut flowers^13^. According to data from Royal Flora Holland, the global distribution center of cut flowers, in 2019, the sales volume of herbaceous peony cut flowers exceeded 72.4 million stems in the Netherlands and >35 million euros, with a year-on-year growth of 20.39% in sales volume and 26.53% in production value^75^. Therefore, there is rapid momentum in the development of the herbaceous peony cut flower market.

## Genetic breeding

### Genetic research

Uncovering genetic mechanisms can serve as an important guide for breeding. Poor self-compatibility^76–80^, high chromosome heterozygosity, and long juvenility have been the greatest limiting factors in *Paeonia* genetic research^81^. In recent years, some scientific institutions have begun to pay attention to *Paeonia*, and some genetic mechanisms are gradually being studied.

#### Trait inheritance

Systematic breeding of *Paeonia* was carried out mainly after the middle of the 18th century. *Paeonia* breeders tend to pay more attention to the compatibility of different cross combinations but pay less attention to the genetic mechanisms underlying those crosses. Combined with the phenotypic characters of the offspring and the parents of previous hybrids, the blotch at the base of the petal and carpel coat covering the tomentum is a dominant inherent character in *Paeonia* breeding^6,67^. In wild peony species, chalconaringenin 2′-*O*-glucoside, which has been detected only in the petals of subsect. *Delavayanae*, can be stably inherited by offspring, resulting in yellow or orange flowers in Lutea and Itoh hybrid peonies^82^. The flower fragrance of subsect. *Delavayanae* species is pleasant, and the main components are linalool compounds, which can be passed on stably to offspring through hybridization^83–85^. Cheng and Chen analyzed the genetic mechanisms of flower color and flower type in hybrid combinations of Northwest tree peonies and found that these traits may be greatly influenced by the female parent and that the inheritance of white flowers and the single flower type is strong (about half of the offspring had white or single flowers)^81^. Zhang et al. analyzed 20 phenotypic characters in an F_1_ genetic population, including 120 individuals, that was obtained by *P. ostii* ‘Fengdan’ (♀) *×* (*P*. × *suffruticosa* ‘Shin Jitsugets-nishiki’) (♂) and found that the variable coefficients of F_1_ individuals’ phenotypic traits ranged from 11.03% to 63.49% and heterosis and transgressive segregation for 20 phenotypic traits^86,87^.

It is necessary to consciously expand the number of hybrid offspring and comprehensively record their phenotypic characters.

#### Genetic map

Given the limitations stated above, namely, high heterozygosity, poor self-compatibility and a long juvenile period, it is difficult to construct an ideal mapping population of *Paeonia*. Thus far, only three genetic populations of *Paeonia* have been cultivated and used to construct genetic maps. Cai et al.^88^ used *P. ostii* ‘FengDanBai’ as the female parent and *P*. × *suffruticosa* ‘HongQiao’, ‘Kaō’ and ‘Kokuryū-nishiki’ as the male parent and constructed three F_1_ segregating populations by artificial pollination. Simple sequence repeat (SSR) markers, which were used to check the three F_1_ populations, revealed that the F_1_ population in a *P. ostii* ‘FengDanBai’ (♀) *×* (*P*. × *suffruticosa* ‘HongQiao’) (♂) cross was a better population for constructing a genetic map of tree peony than the other two populations. Using this F_1_ population, Cai et al.^89^ performed specific-locus amplified fragment (SLAF) sequencing of the F_1_ population to identify markers that could be used to construct a genetic linkage map. Finally, a high-density genetic map of tree peony was constructed, including 1189 SLAF markers in five linkage groups and spanning 920.669 centimorgans (cM). Peng et al.^90^ developed 74 SSR markers with polymorphisms based on the transcriptome data of *P*. × *suffruticosa* ‘LuoYang Hong’. These markers were used to construct a genetic map for the same F_1_ population developed by Cai et al.^89^. A total of 68 SSR loci were mapped to five linkage groups, 48 of which showed Mendelian inheritance (segregation ratio of 1:1, 1:1:1:1 or 1:2:1). By using *P. ostii* ‘Fengdan Bai’ (♀) *×* (*P*. × *suffruticosa* ‘Xin Riyuejin’) (‘Xin Riyuejin’ = ‘Shin Jitsugetsu-nishiki’) (♂), an F_1_ genetic population including 120 individuals was obtained. Zhang et al. used genotyping-by-sequencing (GBS) to develop markers for the Cai et al.^91^ F_1_ population. Finally, a genetic map was constructed containing 3868 markers in five linkage groups with a genetic distance of 13,175.5 cM and 322-1224 markers in each linkage group. Guo et al.^92^ used the same F_1_ population as Zhang et al.^91^ to construct a genetic map by using SSR markers. Finally, 35 SSR markers were mapped, covering five linkage groups, each with 3-14 markers. Li et al.^93^ performed restriction site-associated DNA sequencing (RADseq) of an F_1_ genetic population with 120 individuals, which were derived from (*P*. × *suffruticosa* ‘Qing Long Wo Mo Chi’) (♀) × (*P*. × *suffruticosa* ‘Mo Zi Lian’) (♂) (the two cultivars are from the Zhongyuan tree peony group, with purple-black flowers)^93^. Genetic maps of the parents constructed with RADseq markers showed 1471 markers in the female parent genetic map, in seven linkage groups, and 793 markers in the male parent genetic map, in five linkage groups, covering 965.69 and 870.21 cM, respectively.

The above three genetic populations^89,91,93^ had different parents, but the findings have not been replicated to date. The number of offspring in the three populations was <200, and the phenotypic characters could not be fully separated, which increased the frequency of partial marker separation. The markers mapped to the genetic map are limited, and their distribution is uneven, even though seven linkage groups were included in the female parent genetic map constructed by Li et al.^93^. Zhang et al.^91^ included more molecular markers in their genetic map than in other studies, but the genetic distance that they observed was more than ten times greater than that observed by the other groups, suggesting that additional analyses are needed. Thus far, a limited number of genetic maps have been constructed only for *P*. × *suffruticosa*, with no genetic maps in sect. *Paeonia*. Maps of more and different genetic populations need to be constructed according to their genetic traits.

#### Molecular markers

The origin and history of peony cultivars are incomplete and complicated. Therefore, it is difficult to meet the requirements of modern breeding by the exclusive use of traditional morphological index markers. Compared with traditional morphological index markers, DNA molecular markers have many advantages, such as a high abundance, a large amount of information, and insensitivity to the environment. At present, molecular marker-assisted breeding of *Paeonia* mainly involves two processes: (1) early identification of the authenticity of hybrid seedlings and (2) quantitative trait locus (QTL) mapping of phenotypic traits.

There is a certain amount of self-compatibility in *Paeonia*, and the self-pollinated seed setting rate is <5%^78–80^. During the process of hybridization, improper handling may lead to self-pollination and false hybrids (pollen from unwanted cultivars). Peony seedlings have a long juvenile period (more than 3 years before flowering). To reduce maintenance costs, it is necessary to identify the authenticity of hybrid seedlings early. Several molecular markers, including intersimple sequence repeat (ISSR), amplified fragment length polymorphism (AFLP), sequence-related amplified polymorphism (SRAP), and SSR markers, were used in the early identification of *Paeonia* hybrids (Supplementary Data 4). Different molecular markers have achieved good results in validating the authenticity of and identifying *Paeonia* hybrids^94–103^. Relatively speaking, SSR markers have the advantages of simple operation, high accuracy, good stability, less demand for DNA, and codominance, among others, making them more suitable than other markers for early authenticity validation and identification of *Paeonia* hybrids^97,103^.

There are few genetic populations of *Paeonia*, and QTL research on phenotypic characters in *Paeonia* is very limited. Cai used the F_1_ segregating population and genetic map from *P. ostii* ‘FengDan Bai’ (♀) × (*P*. × *suffruticosa* ‘HongQiao’) (♂) to analyze 27 quantitative characters of branches, leaves, flowers and fruits using composite interval mapping^104^. Among them, 20 traits were successfully detected for associated QTLs, and QTL-pn-2, which controls the number of petals, explained up to 71.9% of the phenotypic variation. In addition, three QTLs associated with flower color traits were detected, explaining 11.4–12.8% of the phenotypic variation.

Wu et al.^101^ used 11 pairs of polymorphic SSR markers to analyze 32 phenotypic characters in a natural population of Northwest tree peonies, showing that it was composed of 99 representative individuals without any direct relationships. They also found that five markers were significantly associated with six traits, each explaining 30.4–55.8% of the phenotypic variation. Wu et al.^102^ used 138 SSR markers to carry out trait association analysis of 462 natural populations and the F_1_ segregating population of *P. ostii* ‘FengDan Bai’ (♀) *×* (*P*. × *suffruticosa* ‘HongQiao’) (♂). In natural populations, SSR markers were significantly correlated with flower, leaf and fruit characteristics. Five associations were confirmed in the F_1_ segregating population, involving four markers and four flower characters: petal width (PS029 and PS296), petal shape (PS029), petal color (PS134), and petal number (PS309). The performance of traits in the above studies^101,102,104^ was assessed at only one location per year, which did not exclude the interference of environmental factors. Therefore, the reliability of these QTLs would be affected, and they still need to be verified.

### Traditional breeding

**Fig. 3:**
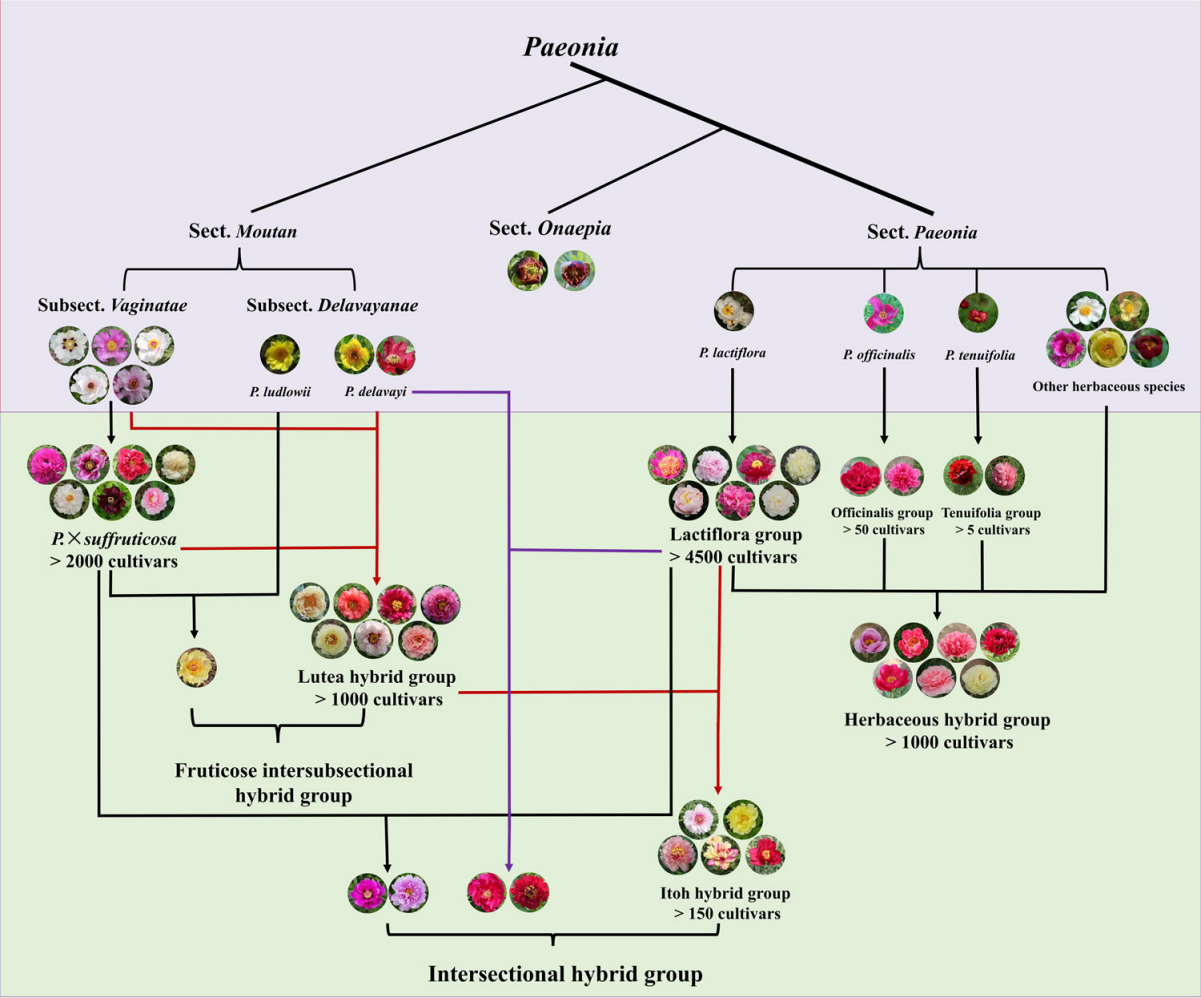
Relationship between wild species and cultivars of *Paeonia*. Wild species and cultivars are distinguished by different background colors, with wild species indicated above and cultivars indicated below. Parents are shown above the arrows while progenies are shown below the arrows. Brackets combine parts of cultivars groups or types into a large cultivar group

Based on 30 *Paeonia* species, more than 8000 cultivars with a rich assortment of flower colors and types have been cultivated through breeding, and the number is growing continuously. Currently, selective breeding and cross-breeding are the most common breeding methods for *Paeonia* (Fig. 3). In addition to these two methods, other techniques have also been used to obtain new *Paeonia* cultivars, such as chemical mutation breeding, radiation mutation breeding, space mutation breeding, and others. The seeds of *Paeonia* are extremely large, and the testa is thick, which limits the penetration of chemical mutagens and rays. Tissue culture is still a challenge in *Paeonia*, and it is difficult to obtain a large number of seedlings through in vitro culture. All these limitations restrict the development of the above breeding techniques. Therefore, their effectiveness has been very limited^11,12,105,106^.

#### Selective breeding

Selective breeding is a simple method in *Paeonia* breeding that focuses mainly on selective breeding of seedlings and bud mutations^5^.

The selective breeding of seedlings is a way to select new cultivars by collecting naturally pollinated seeds that are then sown, and excellent individuals are selected for propagation from the offspring. In China, most of the new *Paeonia* cultivars were selected using this method before 1960. The largest selective breeding program for tree peony seedlings was carried out under the leadership of Professor Hen Yu in Heze from 1956 to 1975, ultimately generating 252 new cultivars^107^. In the 19th and 20th centuries, most of the new Lactiflora peony cultivars bred in Europe and the USA were also obtained by this method, such as ‘Pietertje Vriend Wagenaar’, the 2018 APS Golden Award-winning cultivar^68^. Although this method is simple, sowing seeds and transplanting seedlings require much land, and the workload required to select excellent individuals is very heavy. With the shortage of land and increasing labor cost, this breeding method is gradually being replaced.

Selective breeding via bud mutations involves the discovery of branches with obvious variation and maintaining that variation through asexual propagation. Some *Paeonia* cultivars were obtained in this way, such as *P*. × *suffruticosa* ‘Shima-nishiki’, ‘Hua Er Qiao’ and ‘Ice Age’, the Itoh hybrid peony cultivar ‘White Emperor’, and the Lactiflora peony cultivars ‘Alpine Aire’, and ‘Ann Styer’, among others^6,12,68^.

#### Intrasectional cross-breeding

##### Tree peony

There are two main types of cross-breeding in tree peony: intrasubsectional and intersubsectional.

There is no reproductive isolation between different species (cultivars) in the same subsection of sect. *Moutan*. A large number of viable seeds can be produced by crossing parents in the same subsection, with fertile offspring. In recent years, to enrich the ornamental traits and enhance the resistance of tree peony cultivars, breeders have carried out different types of cross-breeding within subsect. *Vaginatae*, mainly the following three types: crossing different cultivar groups of *P*. × *suffruticosa*, crossing the wild species in subsect. *Vaginatae* with *P*. × *suffruticosa* and crossing different wild species in the same subsection. In this way, a large number of excellent new cultivars have been cultivated, such as *P*. × *suffruticosa* ‘Angel Emily’, ‘Hei Fu Ren’, ‘Wen Hai’, ‘Zi Yu Zui Xue’, and ‘Fei Hua Si Meng’^11,67,68^ (Fig. 3).

Due to obvious reproductive isolation, it is difficult to obtain viable seeds by crossing subsect. *Vaginatae* with subsect. *Delavayanae*^6^. The species of subsect. *Delavayanae* were discovered in the late 19th century, and *P. delavayi* and *P. lutea* were first used for cross-breeding by European breeders. Louis Henry, a French breeder, announced the first intersubsectional-cross cultivar ‘Souvenir de Maxime Cornu’, which was hybridized by M. Maxime Cornu using *P. lutea* (♀) *×* (*P*. × *suffruticosa*) (♂). Victor Lemoine carried out similar work in the same period, and some cultivars had bloomed at ~1900 during World War I. These cultivars quickly caused a sensation after they were displayed in the APS. Most of these cultivars have very heavy flowers, which tend to hang down among the foliage. Inspired by Lemoine, American breeder Arthur Saunders began to introduce his own hybrid tree peonies by using *P. lutea* and *P. delavayi* as the female parents and Japanese *P*. × *suffruticosa* as the male parent. By 1986, a total of 78 cultivars had been registered with the APS. The flower types of these cultivars are mostly single or semidouble, and the flower colors are very rich, from crimson, scarlet, apricot yellow and amber to golden yellow and lemon yellow, while the blooms are held on strong stems well above the leaves^7,9^.

All the F_1_ Lutea hybrid peonies from Europe and Saunders’ collections appeared to be sterile. Accidentally, Saunders obtained two seeds from an F_1_ Lutea hybrid peony (Saunders F2a and Saunders F2b) and managed to develop them into seedlings. The two seedlings were eventually transferred to Nassos Daphnis. Daphnis used these two F_2_ seedlings as important parents to carry out a large amount of backcross breeding and produced a batch of fertile cultivars. Since then, the sterility bottleneck of Lutea hybrid peonies has been broken^9^. In the last 30 years, breeders such as Bill Seidl (USA), Bernard Chow (Australia), and Nate Bremer (USA) have crossed fertile Lutea hybrid peonies (most from Daphnis’ cultivars) with each other, and the fertility of an advanced generation of Lutea hybrid peonies has been greatly improved as a result. The breeding objectives have also shifted, with the focus now on cultivars with fortified vigor, larger flowers born on stronger stems and with good form, such as ‘Alicia Nicole’, ‘Aquarius’, ‘Beach Comber’, and ‘Coral Nebula’^68^.

To improve the adaptability and stem strength of Lutea hybrid peonies, Japanese breeders also attempted to backcross Japanese *P*. × *suffruticosa* with F_1_ Lutea hybrid peonies. Hao et al.^99^ used the F_1_ Lutea hybrid peony ‘High Noon’ as the female parent and some Japanese *P*. × *suffruticosa* cultivars as the male parent in crosses. Finally, five new cultivars were screened out with strong stems and considerable vigor. In China, intersubsectional cross-breeding work in tree peony began at the end of the 20th century and mainly involved crosses of *P. lutea* and *P. delavayi* with *P*. × *suffruticosa*, and new cultivars gradually emerged after 2004, such as ‘Cai Hong’, ‘Xiao Xiang Fei’, and ‘Yan Huang Jin Meng’^11,67,108^ (Fig. 3).

In addition to the cross-breeding combinations mentioned above, breeders have also tried *P*. × *suffruticosa* as the pollen parent, *P. lutea* or *P. delavayi* as the seed parent, or *P. lutea* or *P. delavayi* crosses with wild species of subsect. *Vaginatae* and obtained some cultivars, such as ‘Nong Yuan Xiang Yue’ and ‘Nong Yan Xiang Yu’^9,11,68^. *Paeonia ludlowii* was also used by some Chinese breeders in intersubsectional cross-breeding, but no new cultivars have yet been reported. In April 2019, one of the authors (Yong Yang) visited Heze, Shandong Province, and observed five seedlings of (*P*. × *suffruticosa* ‘Xiang Yu’) (♀) *× P. ludlowii* (♂), which were bred by Xiaojing Zhao, a peony breeder in Heze. The leaf types of these seedlings were intermediate to those of the parents but more inclined towards *P. ludlowii*. Among them, four plants could not form apical buds in winter, and only one flowered, forming single, yellow flowers. This may be the first excellent individual to be obtained by a cross between *P. ludlowii* and a member of subsect. *Vaginatae*.

##### Herbaceous peony

Herbaceous peony breeding mainly includes intra- and interspecific cross-breeding, with the former mainly focusing on *P. lactiflora*. In recent years, herbaceous peony, as a new cut flower, has become popular all over the world, and the mainstream cultivars used as cut flowers are Lactiflora peonies^13^. To meet the demands of the cut flower market, cross-breeding within Lactiflora peony cultivars is still an important direction of herbaceous peony breeding. For example, the excellent *P. lactiflora* cut flower cultivars ‘Shirley Temple’, ‘Mother’s Choice’, and ‘Shirayuki-hime’ are the products of intraspecific cross-breeding of Lactiflora peonies. In addition to *P. lactiflora*, *P. officinalis* and *Paeonia teniflora* have also produced some intraspecific hybrids used for landscaping^68^ (Fig. 3).

To expand the diversity of herbaceous peony, interspecific cross-breeding of sect. *Paeonia* has become a research hot spot in recent years. At the end of the 19th century, European breeders began to cross Lactiflora peonies with local wild herbaceous peony species. The first hybridization was carried out by using *P. lactiflora* (♀) *×Paeonia wittmanniana* (♂), and four varieties were bred. As the most famous herbaceous hybrid peony breeder, Saunders made a pioneering attempt to cross ~28 hybridization groups. Among these groups, Lactiflora peonies generally played a major role in the work of hybridists, serving as parents for over 80% of hybrids^9^. Finally, more than 180 new herbaceous hybrid peony cultivars were screened out^68^. Saunders’ work laid a solid foundation for the development of herbaceous peony hybrids, and cultivars such as ‘Nosegay’, ‘Early Windflower’, ‘Moonrise’, ‘Nova’, and ‘Blushing Princess’ were important parents in later herbaceous peony hybrid breeding. To restore fertility and improve the ornamental value of herbaceous peony hybrids, breeders carried out a large number of cross-breeding trials among their offspring based on Saunders’ work^10,109^. In recent years, a large number of excellent new advanced-generation herbaceous peony hybrid cultivars have been cultivated, such as ‘Salmon Dream’, ‘Lemon Chiffon’, ‘Pastelegance’, and ‘Kim’^68^ (Fig. 3). The seedlings of the hybrids whose fertility has been restored are all tetraploid^10^, but the reason is not clear.

#### Intersectional cross-breeding

The three *Paeonia* sections have great differences in phenotype and ecological habit, and it was once thought that intersectional cross-breeding was impossible. The first intersectional hybrid cultivars of *Paeonia* were created by Taichi Itoh, a Japanese breeder, with the Lutea hybrid peony ‘Alice Harding’ (♀) *× P. lactiflora* ‘Kakohden’ (♂). In 1974, four of the seedlings were registered with the APS by Louis Smirnow and caused an immediate sensation^110–112^. Since then, American breeders have conducted many similar crosses, the three most famous breeders being Don Hollingsworth, Roger Anderson, and Donald Smith^7^. On the basis of their breeding experience, some excellent hybrid parents were also screened out^113,114^. Based on US breeders’ experience, Chinese breeders also cultivated some new Itoh hybrid peony cultivars, such as ‘Jing Gui Mei’, ‘Jing Hua Zhao Xia’, and ‘Huang Die’^11,12,115^ (Fig. 3).

The *P. lactiflora* (♀) *×* Lutea hybrid peony (♂) combination is the most successful combination in *Paeonia* intersectional cross-breeding. To obtain novel varieties, breeders also carried out other types of intersectional cross-breeding. After many failures, some new cultivars were obtained from some new hybrid combinations. The successful cross combinations are as follows: *P. lactiflora* (♀) *×* (*P*. × *suffruticosa*) (♂)^116,117^, *P. lactiflora* (♀) *×P. delavayi* (♂), (*P*. × *suffruticosa*) or Lutea hybrid peony (♀) *×P. lactiflora* (♂)^68,116–118^, and *P. delavayi* (♀) *×P. lactiflora* (♂)^68^ (Fig. 3). In addition to these cross-breeding combinations, breeders have also tried a variety of intersectional cross-breeding combinations, including those involving species of section *Onaepia*. However, only select combinations produced a few seeds, and no new cultivars were reported^119–123^.

Itoh hybrid peonies are generally triploid^124,125^ with poor fertility and produce few effective pollen grains or full seeds. Smith tried to restore the fertility of Itoh hybrid peonies by crossing some of his own Itoh hybrid peony cultivars with Lactiflora peony or an advanced generation of herbaceous hybrid peony cultivars^126^. In the past five years, he made many attempts. Finally, two seedlings were retained (private communication with Don Smith). This attempt may change the status of infertility of Itoh hybrid peonies.

### Molecular breeding

Molecular breeding is a highly efficient way to compensate for the shortcomings of traditional breeding methods and shorten the breeding cycle. At present, the tissue culture of *Paeonia* mainly uses zygotic embryos or buds to produce secondary buds directly^127–130^. It is difficult for *Paeonia* plants to dedifferentiate differentiated tissue to form calli and then form seedlings. This limitation has led to the inability to develop a stable genetic transformation system that is the basis of genetic transformation or gene editing in *Paeonia*. Although there are still many challenges to achieving the molecular breeding of *Paeonia*, the molecular mechanisms of several *Paeonia* characters that are linked with production and practical applications have been researched (Table 2). These molecular studies have laid a foundation for the future molecular breeding of *Paeonia*.

**Table 2:** Genes related to ornamental characters that have been cloned in *Paeonia*

Horticultural traits	Gene
Flower and leaf color	*PlCHS*, *PsCHS1*, *PlCHI*, *PlF3*′*H*, *PlF3H*, *PlFLS*, *PlDFR*, *PsDFR1*, *PsANS*, *PlANS*, *Pl3GT*, *Pl5GT*, *PsAOMT*, *PlPAL*, *PsWD40*, *PsMYB2*
Flower type	*PsAP1*, *PsAP2*, *PlSEP3*, *PlAP3-1*, *PlAP3-2*, *PsTM6*, *PlPI*, *PsPI*, *PsMADS1*, *PsMADS5*, *PsMADS9*, *PsAG*
Flowering time	*PsSOC1*, *PsFT*, *PsSVP*, *PsGA20ox*, *PsCPS*, *PsNCED*, *PSbZIP*, *PsFUL1*, *PsCOL4*
Abiotic stress resistance	*PsGPAT*, *PlDHN2*, *PlHSP70*, *PsPSK1*, *PlTDC*, *PsDREB*, *PlWRKY13*
Post-harvest	*PlACS*, *PsACS*, *PsACO1*, *PlPIP1;2*, *PlPIP2;1*, *PlNIP*, *PlSUT2*, *PlSUT4*, *PlCWIN1*, *PlVIN1*, *PlCIN1*, *PlCIN2*, *PlSPS1*, *PlSPS4*, *PlSUS3*, *PlSUS4*, *PsEIL3*, *PsERF1*
Bud dormancy	*PsPII*, *PsDHN*, *PsGA20*, *PsARP*, *PsCXE*, *PsSERK1*, *PsPOB*, *PsMPT*, *PsGRAS1*, *PsGRAS2*, *PsSERK2*
Seed dormancy	*PoNCED1*, *PoZEP1*, *PoSDR1*, *PsbZIP*, *PsGAI1*

Detailed information of the genes is shown in Supplementary Data 6

#### Flower and leaf color

The main factors affecting flower color are the composition and levels of pigments in petals^131^. The pigments in *Paeonia* petals are flavonoids, including anthocyanins, flavones, and flavonols^82,132–138^. The diversity of flower color is caused by the differential expression of flavonoid biosynthesis-related genes. The metabolic pathway of flavonoids in *Paeonia* is relatively well resolved^139^. The structural genes *DFR* and *ANS* (the full names and details of all genes are provided in Supplementary Data 5 and 6), which play a key role in the synthesis of anthocyanins, determine the formation of pink and red flowers^140–142^, and the high expression of *AOMT* promotes the change in flower color from red to purple^143^. The expression levels of other structural genes, such as *CHS*, *CHI*, *FLS*, *PAL*, *F3H*, *F3’H*, *F3'5*′*H*, *FLS*, *ANR*, *3GT*, *5GT*, *UF3GT*, and *UF5GT*, also play vital roles in yellow, white and other flower colors^144–148^.

In addition to structural genes, transcription factors also play important roles in regulating flower coloration in *Paeonia*. The transcription factors *PsMYB* and *PsWD40* may play a key regulatory role in controlling anthocyanin concentration in red and white petals, and their differential expression mediates the formation of multicolored flowers^142^. *PsMYB12* can activate the *PsCHS* promoter and play a direct role in the formation of tree peony petal blotch, which is when the color at the base of the petal is significantly darker than that of other parts, forming a blotch^148^. *MYB-1* and *MYB-5* may also regulate the formation of tree peony petal blotch^143^. The transcription factor *bHLH3* is highly expressed in white petals of tree peony, while *MYB22* is highly expressed in purple petals. Therefore, these two transcription factors may play a role in regulating the shade of tree peony petals^147^.

The leaf color of most peony cultivars is red when they sprout in spring, giving them ornamental value, but the red gradually fades as the leaves expand. The mechanism of red coloration in *Paeonia* leaves is consistent with that in petals and is also due to the presence of flavonoids. High expression of the structural genes *CHS*, *DFR*, and *ANS* and low expression of *LAR* and *ANR* lead to the accumulation of anthocyanins in red leaves. The anthocyanin repressor *MYB2* is activated in spring, replacing *MYB1*. Regulation of the MYB2 + bHLH1 + WD40-1 complex reduces the expression levels of *CHS*, *DFR*, and *ANS*, leading to a change in leaf color from red to green^149,150^. In the red and nonred leaf stages of *P*. × *suffruticosa* ‘Man Yuan Chun Guang’, the expression of the structural genes *PsDFR* and *PsANS* was the same as that of anthocyanin genes, and the expression levels of *PsFLS*, *PsANR* and flavonol were consistent; moreover, the transcription factors *PsMYB113*, *PsMYB4* and *PsMYBF1* may be important in regulating the expression of these structural genes^151^.

#### Flower type

The flowers of all wild *Paeonia* species are single. After thousands of years of domestication and selection, a rich assortment of flower types has formed, including semidouble, double, Japanese, and bomb. A change in flower type in *Paeonia* mainly depends on a natural increase in petals combined with centripetal or centrifugal petalization of stamens^6^. Research on *Paeonia* flower development revealed that the development of *Paeonia* floral organs is mainly controlled by the MADS-box gene family, and a large number of functional genes have been cloned^152–156^. Shu et al.^157^ cloned *PsTM6* in the euAP3 lineage of tree peony. By analyzing the gDNA sequence of this gene in 23 tree peony cultivars, it was concluded that *PsTM6* might affect the petalization of stamens. Ge et al.^155^ cloned the genes of the MADS-box family related to flower organ development in *P. lactiflora* and analyzed their differences in expression. Their results showed that as the degree of petalization increased, the expression of *PlAP1*, *PlAP2*, and *PlSEP3* also increased, while the expression of *PlAP3-1*, *PlAP3-2*, and *PlPI* decreased. *PlAP1* and *PlSEP3* mainly determine sepals and petals, and *PlAP3-1*, *PlAP3-2*, and *PlPI* mainly determine stamens and petals. *PlAP2* not only determines sepals and petals but also participates in carpel formation. Wu et al. also found a correlation between *PlAP2* and petal formation^153^. Ren cloned and analyzed the expression differences of genes related to flower type in *P*. × *suffruticosa* ‘Zhao Fen’^158^. The results showed that *PsAP1* belonged to the AP1/SQUA subfamily of the MADS-box family, which was mainly expressed in petals, sepals, and carpels, with the highest expression at an early stage of flower bud differentiation. *PsPI*, *PsMADS1*, and *PsMADS9* were mainly expressed in petals and stamens, regulating the formation of petals and stamens. Moreover, *PsAG* transformed into *Arabidopsis thaliana* and *Nicotiana tabacum* also showed that *PsAG* was involved in the regulation of floral organ development^158^. Tang cloned *PsMADS5*, *PsMADS7*, and *PsMADS12* from the petals of *P*. × *suffruticosa* ‘Luo Yang Hong’ and analyzed differences in their expression^150^. Tang’s results suggested that *PsMADS5* was involved in the regulation of calyx development and the petalization of stamens, *PsMADS7* initiated the petalization of stamens and cooperated with multiple members of the MADS-box family to jointly regulate the formation of multiple flower organs, and *PsMADS12* was significantly expressed in the petals of the ‘Lou Zi’ (crown-proliferation) flower type. These three genes may be key genes for stamen petalization.

#### Flowering time

Several pathways related to flowering time have been reported in *A. thaliana*, including the photoperiod, vernalization, gibberellic acid (GA_3_), and autonomic pathways^159^. The transcription factor *PsCOL4*, which is related to flowering time, was cloned from *P*. × *suffruticosa* ‘Zi Luo Lan’. The expression of *PsCOL4* in the stem and leaves was highest at an early stage, and its expression decreased as the flower bud developed, inducing the expression of downstream genes *FT* and *SOC1* to ensure the flowering process^160^. Wang et al.^161^ cloned the MADS-box gene *PsSVP*, which is mainly regulated by GA_3_, into *P*. × *suffruticosa* ‘Luoyang Hong’. The gene was mainly expressed during vegetative growth, with the highest expression in leaves and stems, but its suppressed expression in aborted flower buds was much greater than that in normal flower buds, inhibiting flower formation. The expression level of *AP1* was different among developmental stages in *P. lactiflora* ‘Jinhui’. The expression levels of *AP1* in the inner and outer petals were significantly different, indicating that its expression level might be related to *P. lactiflora* floral organ development^162^. Zhou et al.^163^ isolated *PsFT* from reblooming (*Paeonia×lemoinei* ‘High Noon’) and nonreblooming (*P. suffruticosa* ‘Luo Yang Hong’) cultivars of tree peony. According to differential gene expression analysis and verification of transgenic function, *PsFT* may be closely related to secondary flowering. Wang et al.^164^ analyzed the transcriptome of flower buds of seven different flowering types of tree peony and screened the genes related to secondary flowering, namely, *PsAP1*, *PsCOL1*, *PsCRY1*, *PsCRY2*, *PsFT*, *PsLFY*, *PsLHY*, *PsGI*, *PsSOC1*, and *PsVIN3*. Wei et al.^165^ found that the expression level of *PsFUL1* was highest in flower buds and petals. *PsFUL1* expression in different peony varieties was analyzed, and it was found that expression peaked quickly in early blossoms of *P. × suffruticosa* ‘Luoyanghong’, which promoted the initiation of downstream genes and ensured the occurrence of early blossoms. Therefore, *PsFUL1* may be an important transcription factor in floral transition and flowering regulation.

Some research has also focused on how genes artificially regulate the flowering response. *PdFT*, which was isolated from *P. delavayi*, had the highest level of expression in flower buds, and its expression could be increased by the exogenous application of 1000 mg/L GA_3_ and defoliation and decreased under short sunshine (8-h photoperiod) and low temperature (4 °C). These results indicate that *PdFT* is a key gene in the photoperiodic pathway that determines the switch to flowering^166^. Exogenous GA_3_ induced and inhibited the expression of the gibberellin-related genes *PsCPS* and *PsGA2ox* in tree peony, and defoliation and exogenous GA_3_ inhibited the expression of *PsNCED* and *PSbZIP*, whose expression coincided with the ethylene release curve^167^. *PsGA2ox* is an inhibitory gene for flowering in *P*. × *suffruticosa* ‘Luo Yang Hong’^168^.

#### Abiotic stress resistance

Plants adapt differently to the environment, and their improved resistance to environmental stresses can expand their adaptive area. Research on the abiotic stress resistance of *Paeonia* has mainly focused on plant responses to low versus high temperature, drought, salt, and heavy metals. Under high-temperature (35.75/27.5 °C, average daily maximum/minimum temperature) stress, heat-resistant *P. lactiflora* ‘Zifengyu’ showed enhanced activity of antioxidant enzymes (superoxide dismutase, peroxidase, and catalase) and the expression of *HSP*, which removed reactive oxygen species effectively and prevented plants from dying^169,170^. *HSP70*-overexpressing transgenic plants were tolerant to high temperature^171,172^. In *P. lactiflora*, microRNAs played an important role at the posttranscriptional level: *miR172c-3p*, *miR395a*, *miR397a*, *miR408-5p*, and *miR827* expression was upregulated under high-temperature stress, indicating that they were associated with posttranscriptional regulation under high-temperature stress (35.75/27.5 °C, average daily maximum/minimum temperature)^173^. In salinity stress trials, Hao et al.^174^ cloned *PSK1* from *P*. × *suffruticosa* into Arabidopsis, and transgenic lines formed larger green cotyledons on medium with 125 mM NaCl than wild-type (WT) Arabidopsis. Using transcriptome and miRNA sequencing and comparing the response of *P. ostii* in 100 μM CuSO_4_·5H_2_O copper stress and control groups, 12 genes and 30 miRNAs were preliminarily identified as participating in the regulation of copper stress^175,176^. The tryptophan decarboxylase gene of *P. lactiflora* (*PlTDC*) in the melatonin biosynthesis pathway enhanced the tolerance of transgenic tobacco to drought, which was induced for 18 days in the absence of water at 22°C. When the NaCl concentration was 200 mM, there was no difference between transgenic and WT leaf disks, but when the NaCl concentration was >200 mM, the green area of leaf disks of WT lines gradually shrank, especially at 800 mM, where the WT leaf disks all turned brown, in contrast to those of transgenic lines, in which only a few turned brown^177^. When *P. lactiflora* was treated at a low temperature (4 °C), the expression level of the cold resistance gene *PlGPAT* remained high for 72 h, indicating its role in cold resistance^178^.

The expression level in flower buds of two dehydrin genes, *PlDHN1* and *PlDHN2*, which were cloned from *P. lactiflora* ‘Dafugui’, was highest in December. *PlDHN1* was significantly upregulated in response to heat and waterlogging, and the sensitivity of *PlDHN1* to abiotic stresses was ranked as waterlogging > heat > abscisic acid (ABA) > cold^179,180^. The expression of nucleus-located *PlWRKY13*, which was also cloned from *P. lactiflora* ‘Da Fugui’, was upregulated by four abiotic stresses: low and high temperatures, waterlogging, and salt stress. In addition, *PlWRKY13*-silenced plants were more sensitive to fungal infection, indicating that *PlWRKY13* was induced by abiotic stresses but involved in the regulation of disease resistance^181^. *PsDREB* is a tree peony transcription factor related to stress resistance. Compared with the WT, transgenic lines did not show wilting, the malondialdehyde levels in vivo were lower than those in the WT, and superoxide dismutase and peroxidase were higher than those in the WT, indicating that *PsDREB* significantly improved the resistance of transgenic tobacco to drought and salt stress^182^. Liu et al.^183^ cloned *PsDREB2* and its promoter in tree peony, transferred the promoter into *A. thaliana*, and expressed it in all parts of transgenic plants. Under drought, low-temperature, high-salt, and ABA stresses, the *PsDREB2* promoter upregulated the expression of the stress-related genes *DREB1A*, *CBF1*, *R29A*, and *RD29B*.

#### Postharvest

Since peony flowers have become increasingly popular cut flowers, research on postharvest aspects has also increased. At the molecular level, postharvest research has mainly focused on endogenous hormones, water metabolism and sugar accumulation. Ethylene level is an important factor that affects the preservation of cut flowers. By controlling ethylene content, the quality of cut *Paeonia* flowers can be improved. Zhou et al.^184^ cloned the key genes *PsACS* and *PsACO* involved in ethylene biosynthesis from cut flower petals of *P*. × *suffruticosa* ‘Luoyang Hong’^184^. *PsACS1* was the key gene involved in ethylene biosynthesis in tree peony cut flowers, and *PsACO1* was mainly regulated at the posttranscriptional level during flower opening^184,185^. In addition, glucose relies on the hexokinase signal transduction pathway to inhibit the transcription of *PsACS1*, thus delaying the senescence of tree peony cut flowers^186^. The expression of *PsCTR2* and *PsCTR3* in tree peony increased under the effect of ethylene, and the application of an ethylene signaling inhibitor, 1-MCP, could reverse the process, indicating that *PsCTR2* and *PsCTR3* play a negative regulatory role in response to the ethylene signal^187^. The quality of *P*. × *suffruticosa* ‘Luoyang Hong’ cut flowers was improved by exogenous use of 500 mM glucose, which promoted the expression of *PsCTR3* and inhibited the accumulation of *PsEIL3* transcripts, thereby reducing the sensitivity of cut flowers to ethylene and prolonging the vase life of cut flowers^188^. Subsequent studies also confirmed that the transcription factors *PsEIL3* (triggering transcription of downstream target genes) and *PsERF1* have positive effects on the response to ethylene, playing an important role in the opening and senescence of tree peony cut flowers^188,189^.

Some studies have also been conducted on the genes related to aquaporins and glycometabolism in *Paeonia* postharvest. Zhao et al.^190^ isolated three aquaporin genes, *PlPIP1*;2, *PlPIP2*;1 and *PlNIP*, from *P. lactiflora* ‘Hongyan Zhenghui’ cut flowers. Nanosilver prolonged the vase life of cut flowers, and the expression of *PlPIP1*;2 was higher than that in the control group, while the expression of *PlPIP2*;1 and *PlNIP* was lower than that in the control group, indicating that aquaporins synergistically maintain the water balance of cut flowers. The same effect of aquaporins was also proven by Xue et al.^191^. When adding sucrose (20 g/L) to the vase solution, the flowering duration of *P. lactiflora* ‘Yang Fei Chu Yu’ cut flowers increased by ~1.3 days. Xue et al.^192^ isolated two sucrose transporter genes (*PlSUT2* and *PlSUT4*) and five invertase genes (*PlCWIN1*, *PlVIN1*, *PlCIN1*, *PlCIN2*, and *PlCIN3*) from *P. lactiflora* ‘Yang Fei Chu Yu’: *PlSUT2* and *PlSUT4* were induced by sucrose and played a role in sucrose transport, while *PlCWIN1* and *PlVIN1* were involved in the accumulation of glucose and fructose during the flowering of cut flowers. A study on *P. lactiflora* ‘Yang Fei Chu Yu’ cut flowers showed that the increased expression of *PlSPS1* and *PlSPS4* (coding for starch hydrolase) and the decreased expression of *PlSUS4* (coding for sucrose synthase) led to starch hydrolysis, increased the amount of sucrose in cut flowers and improved the vase quality of cut flowers of *P. lactiflora* ‘Yang Fei Chu Yu’^193^.

#### Bud dormancy

*Paeonia* flower buds typically have internal dormancy. Studies on the mechanism of dormancy in flower buds provide a theoretical basis for artificially breaking dormancy and regulating flowering time. Huang constructed a subtractive cDNA library of the genes related to dormancy release in flower buds of *P*. × *suffruticosa* ‘Lu He Hong’^194^. Eight genes (*PsPII*, *PsDHN*, *PsGA20*, *PsARP*, *PsMPT*, *PsCXE*, *PsSERK1* and *PsPOB*) were screened and may be related to the release from internal dormancy. Based on differential gene expression and transgenic analyses, changes in the expression of *PsARP* may have been related to the transformation between conjugated and free auxin in flower buds, regulating flower bud dormancy at the posttranscriptional level, while *PsMPT* regulated ATP synthesis and promoted the breaking of bud dormancy in tree peony. Zhang et al. obtained the *PsMPT* promoter, and in transgenic plants containing a 421-bp *PSMPT* promoter, the expression and activity of the GUS gene increased significantly after freezing treatment. When the fragment from −421 to −408 base pairs (bp) containing an MYC *cis*-element of the *PSMPT* promoter was deleted, the chilling response was not observed. This result confirmed that the MYC element was a key motif for the chilling response of the *PsMPT* promoter^195^. In other studies, it was also found that *PsPII*, *PsSERK2*, *GA20ox*, *SOC1*, *PsGRAS1*, and *PsGRAS2* may play roles in relieving *Paeonia* flower bud dormancy^196–202^. However, only an analysis of differential expression of genes from different treatments was carried out, and their functions were not further verified by transgenic studies^196–202^.

DNA methylation in flower buds also inhibits the release of dormancy in *Paeonia*. Under the action of exogenous 5-azacytidine (5-azaC), the expression of the DNA methyltransferase genes *PsCMT3*, *PsMET1*, and *PsDRM2* in dormant buds was inhibited, and the transcription of the demethylase gene *PsROS1* increased, thus reducing the DNA methylation level and activating the cell cycle, DNA replication and glycol metabolism processes, which accelerated the dormancy release of buds^203,204^.

#### Seed dormancy

*Paeonia* seeds are dormant, and breaking dormancy is of great importance for improving the reproductive efficiency of *Paeonia*. Research on the molecular mechanisms of seed dormancy in *Paeonia* is scarce, and the main focus has been on the metabolic pathway of hormones. ABA is an important hormone that regulates seed dormancy. During seed development, ABA expression increases first and then decreases^205^. *PoNCED1* and *PoZEP1* may jointly inhibit ABA biosynthesis in *P. ostii* ‘Feng Dan’ seeds. *PoSDR1* induces ABA synthesis in the middle and later stages (104 days to 132 days after pollination). The highest expression of *PobZIP1* in ‘Fengdan’ seeds occurred at an early stage of development (earlier than 90 days after pollination), and the highest ABA content occurred later than the highest expression of *PobZIP1*, suggesting that the transcription factor *PobZIP1* played a negative role in dormancy release^205,206^. The expression of *PlCYP70A1* and *PlCYP70A2*, which are ABA 8′-hydroxylase genes, in the seeds of *P. lactiflora* ‘Fen Yu Nu’ increased during seed germination, promoted ABA degradation, and played a positive role in breaking dormancy^207^. The protein phosphatase gene *PlPP2C* may have a similar mechanism during *P. lactiflora* ‘Fen Yu Nu’ seed germination^208^.

Differential gene analysis was performed on the transcriptome of two developmental stages (0 and 40 days) of *P. lactiflora* ‘Fen Yu Nu’ seeds. The expression of *PlGAI1*, a member of the DELLA protein gene family that regulates gibberellin biosynthesis, was significantly upregulated during seed germination and thus played a positive role in seed germination^209^. Ren et al.^210^ found that the expression of *PoGAPC* associated with glycolysis in *P. ostii* ‘Feng Dan’ seeds continued to be suppressed at a low temperature (4 °C), indicating that delayed glycolysis played a key role in dormancy at low temperatures. Auxin may also play a role in the germination of *P. lactiflora* ‘Fen Yu Nu’ seeds: the expression of the auxin response gene *PlSAUR1* was highest in seeds in the period after germination of the seed hypocotyl, *PlSAUR2* and *PlSAUR3* expression was highest in the period after germination of the seed epicotyl, and *PlSAUR4* expression was highest after seed imbibition^211^.

## Conclusions and perspectives

There are still some doubts pertaining to the taxonomy of *Paeonia*, especially in sect. *Paeonia*. Taxonomists also need to carry out more in-depth and detailed research on *Paeonia*. Due to anthropogenic disturbance, many natural distribution areas of wild species of *Paeonia* are sharply shrinking, and some species are endangered^212,213^. Therefore, conservation efforts are essential. Some wild *Paeonia* species, such as *P. ludowii*, *P. sterniana*, and *P. parnassica*, have relatively strict habitat requirements, and their areas of distribution are very narrow, so the most effective protection strategy for them is in situ conservation. To restore wild populations as soon as possible, artificial pollination and seeding are encouraged, while the harvesting of wild plants for any purpose should be prohibited. Ex situ conservation is also an effective method for protecting rare and endangered species of *Paeonia*, and some significant conservation efforts and achievements have been made in China^67^.

At present, the number of peony cultivars exceeds 8000, but the utilization of wild resources in breeding is very limited. In sect. *Moutan*, *P. decomposita*, *P. rotundiloba*, *P. rockii* subsp. *rockii*, and *P. ludlowii* are rarely used in breeding^46,47^; in sect. *Paeonia*, only approximately seven species (*P. lactiflora*, *P. officinalis*, *P. peregrina*, *P. daurica* subsp. *macrophylla*, subsp. *mlokosevitschii*, *P. tenuifolia*, *P. emodi*, and *P. anomala*) were used in breeding^68^; and in sect. *Onaepia*, no cultivars have been bred. Wild *Paeonia* species are widely distributed around the world, and their phenotypic traits are quite different. The utilization of wild germplasm resources is very important for the development of new cultivars. For example, the use of *P. lutea* and *P. delavayi* allowed for the creation of a new cultivar group, Lutea hybrid peony. In addition, the use of parents from different sections in cross-breeding is still very limited, and a large number of wild species and cultivars have still not been utilized. Future breeding efforts should focus on diversification to enrich the gene pool of peony hybrids.

There are still many deficiencies in *Paeonia* cultivars, such as a relatively narrow flowering period, the unpleasant fragrance of some cultivars, or a limited cultivation range. Therefore, it is necessary to strengthen *Paeonia* breeding by prolonging the flowering period, creating cultivars with a pleasant fragrance and improving the resistance of cultivars to biotic and abiotic stresses.

The propagation of tree peony depends mainly on division and grafting, but propagation efficiency is very low. To improve the propagation efficiency of *Paeonia*, many teams have employed tissue culture, but multiple problems have been encountered: explant browning, difficulties in rooting, bottlenecks in the induction of shoots from callus, and the lack of somatic embryogenic protocols^127–130^. To mass produce *Paeonia* cultivars, embryo rescue of seeds from progeny of distantly hybridized germplasm is required^214,215^. In the future, more attention should be paid to the tissue culture of *Paeonia*, both herbaceous and tree peonies^216,217^.

Production of the current cultivars of *Paeonia* depends almost exclusively on traditional breeding methods, but as modern molecular biotechnology develops, it will be necessary to strengthen and modernize molecular breeding research on *Paeonia*. Many functional genes and transcription factors have been discovered and employed in *Paeonia* research. It is necessary to establish a stable genetic transformation system for *Paeonia* as soon as possible to introduce the functional genes of target characters into homologous plants and to carry out directional breeding. The number of genetic populations of *Paeonia* is very small. Parents with specific traits should be selected to create more abundant genetic populations. QTL mapping or GWASs should be carried out in genetic populations by using multicomponent and performance data to locate the genes and linkage markers associated with target traits. The application of validated markers for specific traits will expedite the breeding process. Many breeding techniques have been applied to model plants and crops, such as magnetic nanoparticle-mediated transgenic technology^218–220^ or carbon nanotube-mediated DNA delivery technology^221^, which can be applied to peony breeding without the need to establish a stable genetic transformation system. The draft genome of *P*. × *suffruticosa* ‘Luo shen xiao chun’ has been published^222^. The first genomic data of *Paeonia* will play a positive role in promoting the modern molecular breeding of members of this genus.

## Supplementary information

Supplementary Data HR
