# Supplementary material for: Germplasm resources and genetic breeding of Paeonia: a systematic review
Source: Hortic Res. 2020 Jul 1;7:107. doi: 10.1038/s41438-020-0332-2 (PMC7327061; doi:10.1038/s41438-020-0332-2)
Supplement: Supplementary file 1 — Supplementary Data HR [file 41438_2020_332_MOESM1_ESM.docx]

**Supplementary Data 1**

***Study design***

A systematic search was carried out in PubMed, ScienceDirect and CNKI by using the key words “peony”, “tree peony”, “herbaceous peony” or “*Paeonia*”, including both English and Chinese language papers in all years. Two independent researchers screened papers related to germplasm resources and genetic breeding based on the titles and abstracts. After reading the full text, original papers that covered the following topics were selected: taxonomy, hybrid breeding, molecular biology of horticultural traits, and genetics. A flowchart summarizing the selection process is presented below.


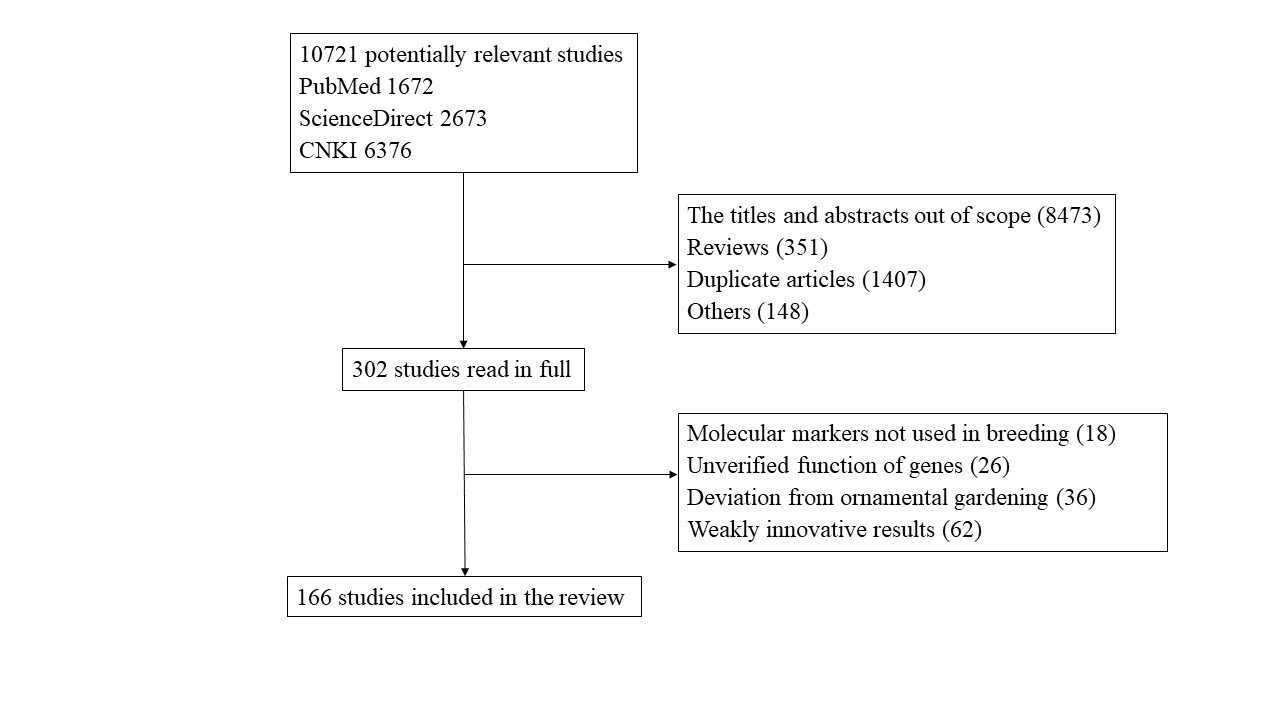


Flowchart of the selection of studies included in the present systematic review.

**Supplementary Data 2**

**Two classical taxonomic views of *Paeonia***

| **Reference** | Stern (1946) | Hong (2010, 2011) |
| --- | --- | --- |
| **I. Section *Onaepia*** | *P. brownie*, *P. californica* | *P. brownie*, *P. californica* |
| **II. Section *Moutan*** | **Subsection *Vaginatae***  *P. suffruticosa* (var. *spontanea*)  **Subsection *Delavayanae***  *P. delavayi*, *P. lutea*, *P. potanini* (var. *trollioides*) | **Subsection *Vaginatae***  *P. cathayana*, *P. decomposita*, *P. rotundiloba*, *P. jishanensis*, *P. ostii*, *P. qiui*, *P. rockii* (subsp. *atava*, subsp. *rockii*), *P.* × *baokangensis*, *P.* × *yananensis*, *P. suffruticosa*  **Subsection *Delavayanae***  *P. delavayi*, *P. ludlowii* |
| **III. Section *Paeon* (*Paeonia*)** | **Subsection Dissectifoliae (four groups)**  Peregrina group: *P. peregrine* Officinalis group: *P. clusii*, *P humilis* (var. *villosa*), *P. officiinalis*, *P. mollis*  Tenuifolia group: *P. tenuifolia* Anomala group: *P. veitchii* (var. *woodwardii*), *P. anomala* (var. *intermedia*)  **Subsection Foliatae (nine groups)**  Lactiflora group: *P. lactiflora* (var. *trichocarpa*), *P. emodi* (var. *glabrata*)  Coriacea group: *P. coriacea* (var. *atlantica*)  Broteri group: *P. broteroi*  Arietina group: *P. arietina* (var. *orientalis*), *P. bakeri*  Mairei group: *P. mairei*, *P. oxypetala*  Obovata group: *P. japonica*, *P. obovata* (var. *willmottiae*)  Mascula group: *P. daurica*, *P. mascula*, *P. banatica*  Russi group: *P. cambessdesii*, *P. russi* (var. *reverchoni*, var. *leiocarpa*), *P. rhodia*, *P. kesrouanensis*  Wittmanniana group: *P. mlokosewitschii*, *P. wittmanniana* (var. *nudicarpa*, var. *macrophylla*) | **Subsection *Albiflorae***  *P. anomala* (subsp. *anomala*, subsp. *veitchii*), *P. emodi*, *P. lactiflora*, *P. sterniana*  **Subsection *Foliatae***  *P. algeriensis*, *P. broteri*, *P. cambessdesii*, *P. clusii* (subsp. *clusii*, subsp. *rhodia*), *P. coriacea*, *P. corsica*, *P. daurica* (subsp. *velebitensis*, subsp. *macrophylla*, subsp. *wittmanniana*, subsp. *mlokosewitschii*, subsp. *daurica*, subsp. *coriifolia*, subsp. *tomentosa*), *P. kesrouanensis*, *P. mairei*, *P. mascula* (subsp. *mascula*, subsp. *russio*, subsp. *bodurii*, subsp. *hellenica*), *P. obovata* (subsp. *obovata*, subsp. *willmottiae*)  **Subsection *Paeonia***  *P. arietina*, *P. intermedia*, *P. parnassica*, *P. peregrine*, *P. saueri*, *P. tenuifolia*, *P. officinalis* (subsp. *microcarpa*, subsp. *banatica*, subsp. *huthii*, subsp. *italica*, subsp. *officinalis*), *P.* × *saundersii* |

**Supplementary Data 3**

***Background information of cultivars (species) in Fig. 2.***

| **No.** | **Taxon** | **Name** | **Origin** |
| --- | --- | --- | --- |
| 1 | *Paeonia rockii* | species | China |
| 2 | *P. mairei* | species | China |
| 3 | Itoh hybrid peony | Scarlet Heaven | USA |
| 4 | Itoh hybrid peony | First Arrival | USA |
| 5 | Itoh hybrid peony | Hillary | USA |
| 6 | Itoh hybrid peony | Prairie Charm | USA |
| 7 | Herbaceous hybrid peony | Coral Sunset | USA |
| 8 | Lactiflora peony | Peter Brand | USA |
| 9 | Lactiflora peony | Miss America | USA |
| 10 | Lutea hybrid peony | Oukan | Japan |
| 11 | Lutea hybrid peony | Hephestos | USA |
| 12 | *P.*×*suffruticosa* | Sai Molian | China |
| 13 | *P.*×*suffruticosa* | Shimadaijin | Japan |
| 14 | Lutea hybrid peony | Xiangfei | China |
| 15 | Itoh hybrid peony | Lollipop | USA |
| 16 | *P.*×*suffruticosa* | Shima-nishiki | Japan |
| 17 | *P.*×*suffruticosa* | Shimane Chōjuraku | Japan |
| 18 | *P.*×*suffruticosa* | Kaō | Japan |
| 19 | Lactiflora peony | Do Tell | USA |
| 20 | Lactiflora peony | Duchesse de Nemours | France |
| 21 | Lactiflora peony | Wladyslava | USA |
| 22 | Lactiflora peony | Angel Cheeks | USA |
| 23 | Herbaceous hybrid peony | Etched Salmon | USA |
| 24 | Lactiflora peony | Laolaihong | China |
| 25 | Herbaceous hybrid peony | Red Charm | USA |
| 26 | Herbaceous hybrid peony | Command Performance | USA |
| 27 | Lutea hybrid peony | Souvenir de Maxime Cornu | France |
| 28 | Lactiflora peony | Fenchi Jinyu | China |
| 29 | Lactiflora peony | Kaguya-hime | Japan |
| 30 | Lactiflora peony | Pietertje Vriend Wagenaar | USA |

**Supplementary Data 4**

***Some molecular markers used for identification of Paeonia hybrids***

| **Molecular marker** | **Parents** | **Description** | **Ref.** |
| --- | --- | --- | --- |
| ISSR | Female parent: *P. ostia*; male parents: *P. suffruticosa* ‘Zhao Fen’ and ‘Zi Er Qiao’ | DNA of two hybrid offspring amplified the specific bands of their parents, confirming that ISSR can be used to identify hybrid offspring at the seedling stage. | 94 |
| AFLP | Female parents: seven *P. suffruticosa* cultivars; male parent: Lutea hybrid peony ‘High Noon’ | From 64 pairs of AFLP primer combinations, nine primer pairs were screened for amplification to identify the nature of 22 offspring from the crosses. Results clearly showed that all were real hybrids, most (86.36%) with the female parent background, and less (13.64%) with the male parent background. | 95, 98 |
| SRAP | Sect. *Peaonia* and sect. *Moutan* | The SRAP Me8/Eml primer pair was used to identify intersectional hybrids. The DNA of intersectional offspring amplified the characteristic bands of parents. | 96 |
| SRAP, ISSR, AFLP, SSR |  | The application of some molecular markers in hybrid breeding was summarized. | 97 |
| SRAP | Female parent: Lutea hybrid peony ‘High Noon’; male parents: *P. suffruticosa* ‘Silver River’, ‘Shou You Den’, and ‘Seppakuzan’ | The SRAP Me8/Eml primer pair was used to detect the parents and five hybrid offspring. The DNA of all the hybrid offspring amplified the characteristic bands of their parents. | 99 |
| SSR | Six wild tree peony species | 14 pairs of SSR markers were used. Zhongyuan and Northwest tree peony groups formed independently by different wild tree peony species. | 100 |
| SSR |  | A total of 11 SSR markers were screened for polymorphism among 99 accessions. Association analysis found five SSR markers associated with six horticultural traits: 30.4% to 55.8% of phenotypic variation could be explained by SSR. | 101 |
| SSR |  | 138 SSR markers were used for trait association analysis of 462 natural populations and the F_1_ segregation population of *P. ostii* ‘FengDan Bai’ (♀)×(*P.* × *suffruticosa* ‘HongQiao’) (♂). Subsequent single-marker association analysis identified 46 significant associations, involving 11 traits with 37 SSR markers. | 102 |
| SSR | Female parents: *P. lactiflora* ‘Fen Yu Nv’, ‘Lian Tai’, ‘Zhu Sha Pan’; male parents: *P. lactiflora* ‘Taff’, ‘Karl Rosenfield’, and herbaceous hybrid peonies ‘Cream Delight’, ‘Roselette’ | 25 suspected hybrids of six combinations were identified by SSR markers: 21 suspected hybrids were shown to be true hybrids. | 103 |

**Supplement Data 5**

***Full name of the genes***

| **Gene abbreviation** | **Full name of the gene** |
| --- | --- |
| *3GT* | 3-*O*-glucosyltransferase |
| *5GT* | 5-*O*-glucosyltransferase |
| *ACO* | ACC oxidase |
| *ACS* | 1-aminocyclopropane carboxylic acid synthase |
| *AG* | agamous |
| *ANR* | anthocyanidin reductase |
| *ANS* | anthocyanidin synthase |
| *AOMT* | anthocyanin *O*-methyltransferase |
| *AP* | apetala |
| *AQP* | aquaporin |
| *ARP* | auxin-repressed protein |
| *bHLH* | a basic helix–loop–helix transcription factor |
| *bZIP* | basic region/leucine zipper motif transcription factor |
| *CBF* | C-repeat binding factor |
| *CHI* | chalcone isomerase |
| *CHS* | chalcone synthase |
| *CIN* | cytoplasmic invertase |
| *CIN* | cytoplasmic invertase |
| *COL* | constans-like |
| *CPS* | *ent*-copalyl diphosphate synthase |
| *CTR* | constitutive triple response |
| *CWIN1* | cell-wall invertase |
| *CXE* | carboxylesterase |
| *CXE* | carboxylase |
| *DFR* | dihydroflavonol 4-reductase |
| *DHN* | dehydrin |
| *DREB* | dehydration-responsive element-binding factor |
| *ERF* | ethylene response factor |
| *F3'5'H* | flavonoid 3’,5’-hydroxylase |
| *F3H* | flavanone 3-hydroxylase |
| *F3'H* | flavonoid 3'-hydroxylase |
| *FLS* | flavonol synthase |
| *FT* | flowering locus T |
| *FUL1* | fruitfull1 |
| *GA2ox* | GA 2-oxidase |
| *GI* | gigantea |
| *GPAT* | glycerol-3-phosphate acyltransferase |
| *HSP* | heat shock protein |
| *LAR* | leucoanthocyanidin reductase |
| *LFY* | leafy |
| *MADS* | MADS-box transcription factor |
| *MPT* | mitochondrial phosphate transporter |
| *MPT* | mitochondrial phosphate transporter |
| *MYB* | myeloblastosis |
| *NCED* | 9-*cis* epoxycarotenoid dioxygenase |
| *NIP* | NOD26-like intrinsic protein |
| *PAL* | phenylalanine ammonialyase |
| *PI* | pistillata |
| *PIP* | plasma membrane intrinsic protein |
| *PSK* | S-phase kinase-associated protein1 |
| *SAUR* | small auxin up RNA |
| *SDR* | short-chain dehydrogenase / reductase |
| *SEP* | sepallata |
| *SERK* | somatic embryogenesis receptor kinase |
| *SOC* | suppressor of constans of overexpression1 |
| *SPS* | sucrose phosphate synthase |
| *STK* | seedstick |
| *SUS* | sucrose synthase |
| *SUT* | sucrose transporter |
| *SVP* | short vegetative phase |
| *TDC* | tryptophan decarboxylase |
| *UF3GT* | UDP-glucose:flavonoid 3-*O*-glucosyltransferase |
| *UF5GT* | UDP-glucose:flavonoid 5-*O*-glucosyltransferase |
| *VIN* | vernalization insensitive |
| *VIN1* | vacuolar invertase |
| *WD* | β-propeller protein group |
| *WIN* | wall invertase |
| *WRKY* | WRKY family members |
| *ZEP* | zeaxanthin epoxidase |

**Supplement Data 6**

**Genes related to ornamental characters that have been cloned in *Paeonia***

| **Gene** | **Description** | **Function** | **GenBank Accession No.** | **Reference #** |
| --- | --- | --- | --- | --- |
| *PlCHS* | chalcone synthase | Structural gene of flavonoid biosynthesis pathway | JN132108 | 142 |
| *PsCHS1* | chalcone synthase | Structural gene of flavonoid biosynthesis pathway | KJ466964 | 139 |
| *PlCHI* | chalcone isomerase | Structural gene of flavonoid biosynthesis pathway | JN119872 | 138 |
| *PlF3’H* | flavonoid 3'-hydroxylase | Structural gene of flavonoid biosynthesis pathway | JQ070803 | 141 |
| *PlF3H* | flavanone 3-hydroxylase | Structural gene of flavonoid biosynthesis pathway | JQ070802 | 141 |
| *PlFLS* | flavonol synthase | Structural gene of flavonoid biosynthesis pathway | KM259902 | 138 |
| *PlDFR* | dihydroflavonol 4-reductase | Structural gene of flavonoid biosynthesis pathway | JQ070804 | 141 |
| *PsDFR1* | dihydroflavonol 4-reductase | Structural gene of flavonoid biosynthesis pathway | KJ466968 | 139 |
| *PsANS* | anthocyanidin synthase | Structural gene of flavonoid biosynthesis pathway | KM871194 | 144 |
| *PlANS* | anthocyanidin synthase | Structural gene of flavonoid biosynthesis pathway | JQ070805 | 141 |
| *Pl3GT* | flavonol 3-*O*-glucosyltransferase | Structural gene of flavonoid biosynthesis pathway | JQ070806 | 141 |
| *Pl5GT* | flavonol 5-*O*-glucosyltransferase | Structural gene of flavonoid biosynthesis pathway | JQ070807 | 141 |
| *PsAOMT* | anthocyanin, *O*-methyltransferase | Structural gene of flavonoid biosynthesis pathway | FE529149 | 140 |
| *PlPAL* | phenylalanine ammonia-lyase | Flavonoid biosynthesis structural gene | JQ070801 | 138 |
| *PsWD40* | WD40 protein | Transcription factor of flavonoid biosynthesis pathway | KJ466974 | 139 |
| *PsMYB2* | MYB transcription factor | Transcription factor of flavonoid biosynthesis pathway | KJ466975 | 147 |
| *PsAP1* | apetala 1 | Gene related to flower organ development (sepals and petals) | HM143943 | 155 |
| *PsAP2* | apetala 2 | Gene related to flower organ development (sepals, petals and carpels) | HQ222889 | 155 |
| *PlSEP3* | PLSEP3-638 MADS box transcription factor SEPALLATA3 | Gene related to flower organ development (sepals, petals and carpels) | KU613325 | 152 |
| *PlAP3-1* | transcription factor APETALA3-1 | Gene related to flower organ development (stamens and petals) | KU613252 | 152 |
| *PlAP3-2* | transcription factor APETALA3-2 | Gene related to flower organ development (stamens and petals) | KU613268 | 152 |
| *PsTM6* | MADS box transcription factor TM6 | Gene related to flower organ development (stamens and petals) | HQ222890 | 154 |
| *PlPI* | pistillata | Gene related to flower organ development (stamens and petals) | KC354379 | 152 |
| *PsPI* | pistillata | Gene related to flower organ development (stamens and petals) | HQ878444 | 155 |
| *PsMADS1* | MADS box transcription factor TM6 | Gene related to flower organ development | HQ222890 | 155 |
| *PsMADS5* | MADS-box protein 5 | Gene related to flower organ development (stamens and sepals) | HQ449569 | 155 |
| *PsMADS9* | MADS9 | Gene related to flower organ development | HQ902183 | 155 |
| *PsAG* | agamous | Gene related to flower organ development | HQ222891 | 155 |
| *PsSOC1* | CONSTANS 1 | Flowering regulation, secondary flowering | KC493630 | 159 |
| *PsFT* | FT | Photoperiodic pathway of flowering regulation, secondary flowering | KF113360 | 159 |
| *PsSVP* | SVP | Gibberellin-pathway related gene in flowering regulation | KC847164 | 159 |
| *PsGA20ox* | GA20ox | Gibberellin-pathway related gene in flowering regulation | MH546118 | 165 |
| *PsCPS* | ent-copalyl diphosphate synthase | Gibberellin-pathway related gene in flowering regulation | MH546115 | 164 |
| *PsNCED* | 9-*cis*-epoxycarotenoid dioxygenase | Gibberellin-pathway related gene in flowering regulation | MH510239 | 164 |
| *PSbZIP* | basic region/leucine zipper motif transcription factor | Gibberellin-pathway related gene in flowering regulation | MH510240 | 164 |
| *PsFUL1* | AP1/FUL-like protein | Transcription factor in flowering regulation and flowering transition | KX621277 | 162 |
| *PsCOL4* | CONSTANS 4 | Transcription factor in flowering regulation | KF113358 | 157 |
| *PsGPAT* | 2 glycerol-3-phosphate acyltransferase | Low temperature resistance | AY016275 | 175 |
| *PlDHN1* | SK3 dehydrin | Low temperature resistance | KX524516 | 177 |
| *PlDHN2* | dehydrin 2 | Waterlogging resistance | KY272747 | 177 |
| *PlHSP70* | heat shock protein 70 | High temperature resistance | JN180465 | 167 |
| *PsPSK1* | a SKP1-like gene homologue | Salt resistance | FE529999 | 170 |
| *PlTDC* | tryptophan decarboxylase | Melatonin biosynthesis pathway gene, resistance to drought and salt stress | KY765554 | 174 |
| *PsDREB* | dehydration responsive element binding protein | Resistance to drought and high salt | KX121328 | 180 |
| *PlWRKY13* | WRKY transcription factor 13 | Disease resistance | KY271095 | 178 |
| *PlACS* | 1-aminocyclopropane-1-carboxylate synthase-like | Ethylene biosynthesis pathway gene in cut flowers | JN639532 | 187 |
| *PsACS* | 1-aminocyclopropane-1-carboxylate synthase | Ethylene biosynthesis pathway gene in cut flowers | DQ337250 | 181 |
| *PsACO1* | 1-aminocyclopropane-1-carboxylate oxidase | Ethylene biosynthesis pathway gene in cut flowers | DQ337251 | 181 |
| *PlPIP1;2* | PIP1;2 | Aquaporin gene | KX235309 | 187 |
| *PlPIP2;1* | PIP2;1 | Aquaporin gene | KX235310 | 187 |
| *PlNIP* | NIP | Aquaporin gene | KX235312 | 187 |
| *PlSUT2* | sucrose transporter (SUT2) | Sucrose transport gene | MG812502 | 189 |
| *PlSUT4* | sucrose transporter (SUT4) | Sucrose transport gene | MG812503 | 189 |
| *PlCWIN1* | cell-wall invertase (CWIN1) | Accumulation of glucose and fructose in cut flowers | MG812500 | 189 |
| *PlVIN1* | vacuolar invertase (VIN1) | Accumulation of glucose and fructose in cut flowers | MG812501 | 189 |
| *PlCIN1* | cytoplasmic invertase (CIN1) | Accumulation of glucose and fructose in cut flowers | MG812497 | 189 |
| *PlCIN2* | cytoplasmic invertase (CIN2) | Accumulation of glucose and fructose in cut flowers | MG812498 | 189 |
| *PlSPS1* | sucrose phosphate synthase 1 | Starch hydrolysis in cut flowers | MK411474 | 190 |
| *PlSPS4* | sucrose phosphate synthase 4 | Starch hydrolysis in cut flowers | MK411475 | 190 |
| *PlSUS3* | sucrose synthase 3 | Starch hydrolysis in cut flowers | MK411476 | 190 |
| *PlSUS4* | sucrose synthase 4 | Starch hydrolysis in cut flowers | MK411477 | 190 |
| *PsEIL3* | ethylene insensitive 3-like 3 protein | Transcription factor that responds to ethylene signals | JQ771471 | 185 |
| *PsERF1* | ERF transcription factor | Transcription factor in the ethylene signal transduction pathway | KR527267 | 186 |
| *PsPII* | PII protein | Gene related to dormancy release in flower bud | EU072920 | 191, 194 |
| *PsDHN* | dehydrin 1-like protein | Gene related to dormancy release in flower bud | EU091154 | 191 |
| *PsGA20* | GA 20-oxidase | Gene related to dormancy release in flower bud | EF607139 | 191 |
| *PsARP* | 51 auxin-repressed protein | Gene related to dormancy release in flower bud | EU072919 | 191 |
| *PsCXE* | 199 CXE carboxylesterase | Gene related to dormancy release in flower bud | EU072921 | 191 |
| *PsSERK1* | 527 somatic embryogenesis receptor kinase | Gene related to dormancy release in flower bud | EU072923 | 191 |
| *PsPOB* | 11 putative protein mRNA, partial cds. | Gene related to dormancy release in flower bud | EF608939 | 191 |
| *PsMPT* | 37 mitochondrial phosphate transporter | Regulation of ATP synthesis and promotion of dormancy release of flower bud | EU072922 | 191 |
| *PsGRAS1* | gibberellin-insensitive 1 | Transcription factor related to bud dormancy | MH061319 | 199 |
| *PsGRAS2* | gibberellin-insensitive 1 | Transcription factor promoting bud dormancy release | MH061320 | 199 |
| *PsSERK2* | somatic embryogenesis receptor-like kinase | Gene related to dormancy release of buds induced by low temperature | KY200849.1 | 195 |
| *PoNCED1* | 9-*cis*-epoxycarotenoid dioxygenase | Inhibition of ABA biosynthesis in seeds | KM405433 | 202 |
| *PoZEP1* | zeaxanthin epoxidase | Inhibition of ABA biosynthesis in seeds | KM405432 | 202 |
| *PoSDR1* | short-chain dehydrogenase / reductase | Induction of ABA biosynthesis in seeds | KM405434 | 202 |
| *PsbZIP* | basic region/leucine zipper motif transcription factor | Transcription factor associated with the ABA signaling pathway in seed | MH510240 | 203 |
| *PsGAI1* | gibberellin-insensitive 1 | Transcription factor associated with gibberellin biosynthesis in seeds | MH550803 | 206 |
